# Profiling of core fucosylated N-glycans using a novel bacterial lectin that specifically recognizes α1,6 fucosylated chitobiose

**DOI:** 10.1038/srep34195

**Published:** 2016-09-28

**Authors:** Saulius Vainauskas, Rebecca M. Duke, James McFarland, Colleen McClung, Cristian Ruse, Christopher H. Taron

**Affiliations:** 1 New England Biolabs, 240 County Road, Ipswich, MA 01938, USA; 2 Charles River Laboratories, 8 Henshaw St., Woburn, MA 01801, USA

## Abstract

A novel fucose-binding lectin (SL2-1) from the bacterium *Streptomyces rapamycinicus* was identified by analysis of metagenomic DNA sequences. SL2-1 belongs to a new group of bacterial fucose-specific lectins that have no similarity to known bacterial fucose-binding proteins, but are related to certain eukaryotic fucose-binding lectins. The 17 kDa protein was expressed recombinantly in *E. coli* and purified by affinity chromatography. Glycan microarray analysis with fluorescently labeled recombinant SL2-1 demonstrated its ability to bind to core α1-6 fucosylated N-glycans, but not to core α1-3 fucosylated N-glycans, or other α1-2, α1-3 and α1-4 fucosylated oligosaccharides. The minimal high affinity binding epitope of SL2-1 was α1-6 fucosylated di-n-acetylchitobiose. The recombinant lectin was efficient in detection of N-glycan core fucosylation using lectin blotting and lectin ELISA assays. Finally, a workflow using SL2-1 for selective and quantitative profiling of core fucosylated N-glycans using UPLC-HILIC-FLR analysis was established. The approach was validated for selective capture and analysis of core fucosylated N-glycans present in complex glycan mixtures derived from mammalian serum IgG.

Fucosylation of N-glycans occurs throughout eukaryotes and plays an important role in a variety of cellular processes, including signaling, cell adhesion, fertilization and cell growth^1^,^2^,^3^. Heterogeneity of fucosylation, such as the number of fucose residues and their linkages, depends on the organism as well as on the physiological conditions of a cell. Mammalian N-glycans may contain fucose in multiple locations. Fucose residues are often found in an α1,6-linkage on the innermost GlcNAc residue of the N-glycan chitobiose core, referred to as ‘core’ fucose. It may also be present in an α1,2-, α1,3-, or α1,4-linkage on galactose residues on the outer arms of N-glycans.

Core α1-6 fucosylation is a common modification of mammalian N-glycans. Altered levels of core fucosylation have been observed in certain types of diseases, such as hepatocellular carcinoma, gastric, pancreatic, prostate and colorectal cancers^4^,^5^,^6^. Additionally, the absence of core α1-6 fucosylation on N-glycans of certain anti-cancer therapeutic antibodies affects the efficiency to which they bind to Fc receptors on lymphocytes and induce antibody dependent cell cytotoxicity (ADCC)^7^.

A molecular tool that discriminates between core and outer arm fucosylation, and that permits highly specific detection and quantification of core fucose is needed. Such a reagent would permit a better understanding of the biological role of core fucosylated N-glycans and would be useful in the identification of potential glycan based biomarkers of disease. Most known core fucose-binding lectins do not discriminate between core and outer arm N-glycan fucosylation and may bind to other carbohydrate residues^8^,^9^,^10^,^11^. Recently, several small lectins of fungal and algal origin have been reported to have stringent specificity for core α1-6 fucose, but they have not successfully been produced as recombinant proteins or they were not able to bind certain core fucosylated structures^12^,^13^,^14^,^15^, thus limiting their utility in glycan analysis strategies.

In the present study, we report identification and characterization of a protein from *Streptomyces rapamycinicus* with a previously unknown function. We show that this protein specifically binds to α1-6 fucosylated chitobiose and defines a new group of fucose-specific lectins found in actinomycete bacteria. The lectin is abundantly expressed in *E. coli*. The purified recombinant protein showed strict specificity and strong binding affinity for a variety of mammalian core α1-6 fucosylated N-glycans. The recombinant lectin was tested and validated in various applications for the detection and quantification of core α1-6 fucosylation on glycoproteins. Finally, efficient use of SL2-1 for epitope-specific profiling of core fucosylated N-glycans present in complex mixtures of mammalian glycans was demonstrated.

## Results

**Figure 1:**
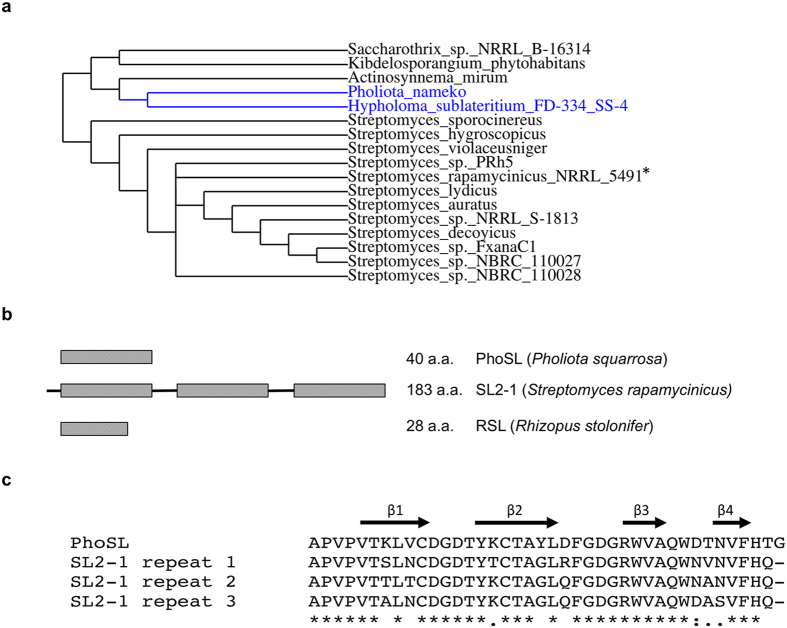
Phylogeny, structural organization and internal sequence homology of SL2-1. (**a**) A maximum-likelihood phylogenetic tree of identified fucose-binding proteins. A phylogenetic tree was generated from the full-length amino acid sequences of the identified proteins. The tree was constructed using a phylogeny analysis program with the default parameters (rooting at mid-point) at www.phylogeny.fr^29^. Eukaryotic proteins are labeled in blue and bacterial proteins – in black. An asterisk indicates the selected candidate protein SL2-1 analyzed in this study. (**b**) SL2-1 is organized in triplicate repeats of a short fucose-binding motif. It represents the typical organization of the putative lectins identified in this study. The domain organization of the PhoSL and RSL lectins used as search queries is also presented for comparison. (**c**) Alignment of PhoSL and the repeat regions of SL2-1. Each repeat domain consists of a predicted four-stranded antiparallel β-sheet.

Identification of candidate bacterial fucose binding lectins was conducted by similarity searching using the BLAST algorithm to query metagenomic sequence databases. The protein sequences of two highly similar fungal lectins, RSL from *Rhizopus stolonifer*^12^ and PhoSL from *Pholiota squarrosa*^14^, were used as search queries. These small fungal oligopeptides each possess an ability to bind to core α1-6 fucose on N-glycans^12^,^14^. The search identified a group of hypothetical proteins of previously unknown function from Gram-positive bacteria (Fig. 1a) from different families of actinomycete bacteria (*Actinosynnemataceae*, *Pseudonocardiaceae* and *Streptomycetaceae*), with most candidates being found in *Streptomycetaceae*. In addition, hypothetical proteins from the basidiomycete fungi *Pholiota nameko* and *Hypholoma sublateritium* were identified.

The identified bacterial proteins each have a similar length (159–188 a.a.) and are organized in triplicate repeats of a putative ~40 a.a. fucose-binding motif comprising the fungal PhoSL sequence (Fig. 1b). The repeating domains within each protein were highly similar (80–90% homology), but not identical (Fig. 1c). Additionally, the similarity between the repeats from different bacterial proteins was also high (60–90%) (see Supplementary Fig. S1). The identified proteins displayed no amino acid sequence similarity to known bacterial fucose-binding lectins from *Ralstonia solanacearum*, *Burkholderia ambifaria* or *Pseudomonas aeruginosa*, that have a six-bladed β-propeller structure^8^,^16^,^17^, or to F-type lectins^18^. These observations suggest that the proteins identified in our searches comprise a new bacterial fucose binding lectin family. Thus, to investigate the glycan binding specificity of a member of this protein family, the hypothetical protein from *Streptomyces rapamycinicus* NRRL 5491 was selected for further study and was designated SL2-1.

### Expression and purification of the SL2-1 lectin

The deduced SL2-1 protein sequence contains a signal peptide as predicted by the SignalP algorithm^19^, suggesting that it is normally a secreted protein. Therefore, for expression in *E. coli*, a codon-optimized DNA sequence encoding a methionine start codon and the SL2-1 coding region (from amino acids 26–183) was synthesized. Recombinant SL2-1 was abundantly expressed in *E. coli* and was purified by affinity chromatography using α-L-fucose agarose resin (see Supplementary Fig. S2). No binding of recombinant SL2-1 to *N*-acetylglucosamine, *N*-acetylgalactosamine or mannose conjugated agarose affinity resins was observed. Purified SL2-1 migrated as a single 17 kDa band via reducing SDS-PAGE, consistent with its calculated molecular weight of 16.86 kDa (see Supplementary Fig. S2). Additionally, electrospray ionization mass spectrometry of recombinant SL2-1 yielded a molecular mass of 16.72 kDa, corresponding to the protein lacking its initiating methionine (see Supplementary Fig. S2). The apparent molecular mass of the native SL2-1 was estimated to be 16.1 kDa by gel filtration suggesting that SL2-1 functions as a monomer (see Supplementary Fig. S2).

### Determination of the glycan binding specificity of SL2-1

The binding specificity of SL2-1 was determined by assessing its ability to bind to different glycoconjugates using two different binding assays. A total of 56 oligosaccharide substrates (see Supplementary Table S1) that had been reductively labeled with the fluorophore 7-amino-4-methylcoumarin (AMC) were used in an immobilized SL2-1 binding assay. In this experiment, biotinylated SL2-1 was coupled to streptavidin coated microtiter plates and incubated with each AMC-glycan. After thorough washing, binding was quantified via fluorescence measurement at 366 nm.

**Figure 2:**
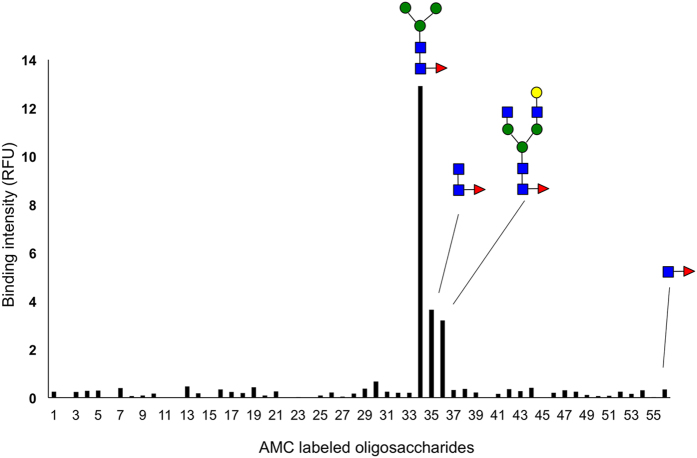
Microtiter plate glycan binding screening assay. Biotinylated SL2-1 was coupled to a streptavidin coated microtiter plate. The specificity of SL2-1 was determined by testing different AMC-labeled carbohydrates for binding to the immobilized lectin (see Supporting Table S1 for the tested carbohydrate structures).

Of the 56 oligosaccharides tested, only three structures bound to SL2-1 (Fig. 2); a core α1-6 fucosylated trimannosyl N-glycan, a core α1-6 fucosylated complex N-glycan and α1-6 fucosylated chitobiose, the conserved core of all N-glycan species. Interestingly, SL2-1 did not bind to a Fucα1-6GlcNAc disaccharide, suggesting that the minimal high affinity epitope of SL2-1 is α1-6 fucosylated chitobiose. No binding was observed for high mannose or complex N-glycans lacking α1-6 fucose or O-glycans bearing α1-2, α1-3 or α1-4 fucose. Additionally, no binding to α1-3 fucosylated N-glycans, a modification found in plants and insects, was observed when the binding assay was performed using immobilized fluorescein-labeled glycopeptides derived from horseradish peroxidase (HRP) (data not shown).

**Figure 3:**
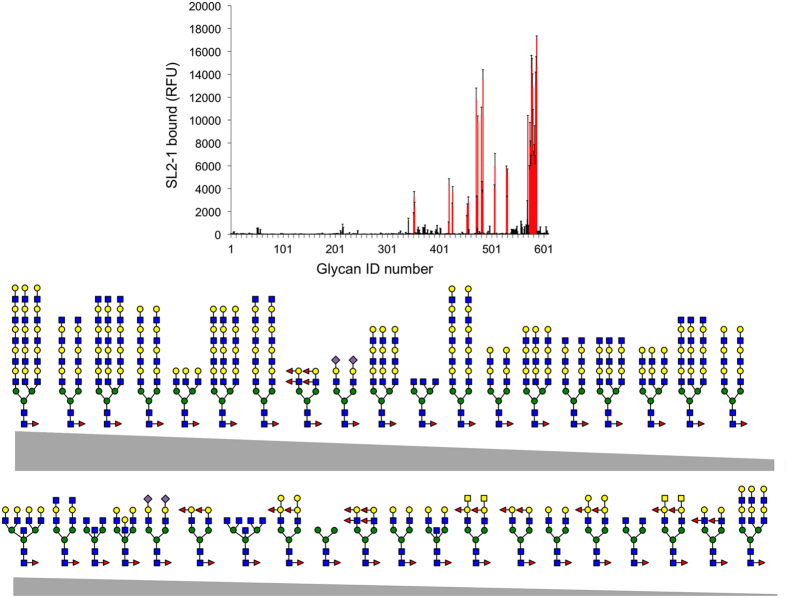
Carbohydrate binding specificity of SL2-1 using a mammalian glycan array. Fluorescent dye-labeled SL2-1 (200 μg/mL) was used for glycan array screening at the Protein-Glycan Interaction Core H of the Consortium for Functional Glycomics (Emory University, Atlanta, GA). The specificity of SL2-1 was determined by testing its ability to bind to a printed array (version 5.2) consisting of 609 mammalian glycans. Of these, 40 structures contained core α1,6 fucose (upper panel, shown in red). The structures of bound core α1,6 fucosylated N-glycans are shown in decreasing order of ligand binding efficiency (see Supporting Table S2 for more details).

The glycan binding specificity of SL2-1 was further explored using a mammalian glycan array at the Consortium for Functional Glycomics (CFG). The array (variant 5.2) consisted of 609 natural and synthetic mammalian glycans with amino linkers printed onto N-hydroxysuccinimide (NHS)-activated glass microscope slides. Of the 609 array glycans, 199 contain either α1-2, α1-3, α1-4, or α1-6 fucose. Additionally, 40 of these structures were core α1-6 fucosylated N-glycans. The array was probed with DyLight 488 labeled SL2-1 as described in Materials and Methods. Interestingly, SL2-1 bound exclusively to core α1-6 fucosylated N-glycans (Fig. 3), showing strong positive binding to 35 structures as determined using a universal threshold method^20^. This approach defines positive binders for any specific lectin as glycans for which the average fluorescence was >10% of the maximum signal observed for that lectin. SL2-1 displayed somewhat weaker binding to 4 structures, for which the average fluorescence was 5–10% of the maximum signal observed. Only one core α1-6 fucosylated N-glycan showed no significant binding (Supplementary Table S2).

SL2-1 bound bi-, tri- and tetraantennary α1-6 fucosylated N-glycans, N-glycans containing bisecting GlcNAc, and N-glycans containing sialic acids. The strongest binding was to core α1-6 fucosylated N-glycans containing a mono- or polylactosamine motif. Even though the binding assay was performed using a very high concentration of the lectin (200 μg/mL), no significant binding to the core afucosylated N-glycans containing a mono- or polylactosamine motifs, α1-2, α1-3, or α1-4 fucose on outer chains, or to the other fucosylated oligosaccharides in the array was detected, further suggesting a strict specificity for the core α1-6 fucose of N-glycans.

Considered together, these data strongly support the conclusion that SL2-1 is a bacterial lectin with high selectivity for binding N-glycans having α1-6 fucosylation of the chitobiose core. Additionally, our data suggest that GlcNAcβ1-4(α1-6Fuc)GlcNAc is a minimal high affinity epitope for SL2-1.

### Determination of binding affinity by titration microcalorimetry

Isothermal titration calorimetry (ITC) was used to assess the strength of SL2-1 binding to various fucosylated glycans. Two ligands were used for the study: α1-6 fucosylated chitobiose (N2F) and a monosialo-fucosylated biantennary N-glycan (A1F) (see Supplementary Fig. S3). An independent model was used to fit the titration data and the calculated thermodynamic parameters are listed in Table 1. The inflection point of the titration curves indicated a stoichiometry of ligand/protein binding that was close to 2, suggesting that there are likely two binding sites per protein. SL2-1 showed a high affinity for both N2F (*K*_*d*_ = 1.59 × 10^−6 ^M) and the complex N-glycan A1F (*K*_*d*_ = 1.06 × 10^−6 ^M). The thermodynamics indicate that the binding of both oligosaccharides is driven by enthalpy. N2F and A1F have similar strong enthalpies of binding (*ΔH* values of −46.36 and −49.18 kJ/mol, respectively), which are in part offset by unfavorable entropies (*TΔS* values of −13.25 and −15.06 kJ/mol).

### Detection of N-glycan core fucosylation on glycoproteins

The strict selectivity of SL2-1 for core α1-6 fucosylated N-glycans suggests that it could be used as a reagent in a number of different applications involving detection or quantification of core fucose on glycoproteins. Therefore, we used SL2-1 in both lectin blotting and lectin ELISA assays to demonstrate its feasibility for detecting the core fucose epitope on mammalian glycoproteins.

**Figure 4:**
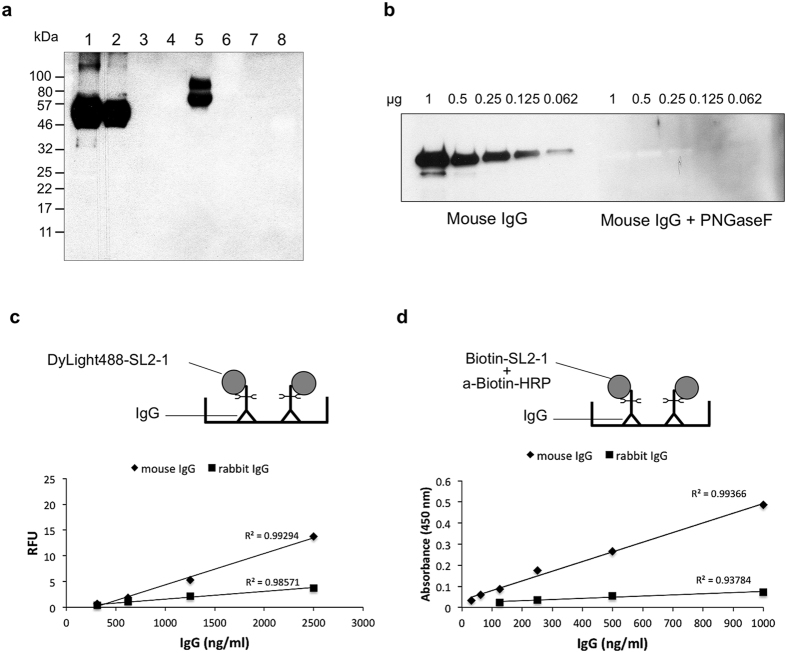
Detection of core fucosylation by lectin blot and lectin-based ELISA. (**a**) Biotinylated SL2-1 (0.6 μg/mL) was used for lectin blotting with different glycosylated and non-glycosylated proteins: 2 μg of mouse IgG (lane 1); 1 μg of mouse IgG (lane 2); 2 μg of mouse IgG treated with PNGase F (lane 3); 2 μg of mouse IgG treated with Endo S (lane 4); 1.5 μg of native CIP (lane 5); 1.5 μg of recombinant CIP expressed in yeast (lane 6); 2 μg of BSA (lane 7); 2 μg of horseradish peroxidase (lane 8). After incubation with the lectin, the membrane was incubated with an HRP-conjugated anti-biotin antibody, and targets visualized using chemiluminescent substrate. (**b**) Titration of murine IgG and detection by SL2-1 lectin blotting. (**c,d**) SL2-1 was tested in a direct lectin ELISA assay that utilized a microtiter plate with immobilized IgG and fluorescent dye-labeled lectin (**c**) or biotin-labeled lectin together with HRP conjugated biotin antibody and TMB colorimetric substrate (**d**) to quantify core fucosylation of IgG molecules.

Biotinylated SL2-1 was used for lectin blotting to probe different glycosylated and non-glycosylated proteins. In this assay, a target glycoprotein was separated by SDS-PAGE, blotted to nitrocellulose, and probed with biotinylated SL2-1. To visualize bound lectin, the blot was further probed with a horseradish peroxidase (HRP) conjugated anti-biotin antibody. Because more than 97% of murine IgG N-glycans contain core α1-6 fucose^21^, the ability of SL2-1 to bind the N-glycans of a model mouse monoclonal IgG2A antibody (anti-MBP) was assessed. SL2-1 bound efficiently to the anti-MBP heavy chain (Fig. 4a, lanes 1–2), but no binding was observed for PNGase F- or Endo S-treated mouse IgG (Fig. 4a, lanes 3 and 4, respectively), indicating that the interaction of SL2-1 and anti-MBP was N-glycan-dependent. Additionally, calf intestinal phosphatase (CIP), an enzyme previously shown to have N-glycans containing core α1-6 fucose^22^, was bound by SL2-1 (Fig. 4a, lane 5), but no binding was observed for yeast-expressed recombinant CIP (that contains high-mannose N-glycans, lane 6), bovine serum albumin (which naturally lacks N-glycans, lane 7), or horseradish peroxidase (that contains core α1-3 fucosylated N-glycans, lane 8).

These results again illustrate the high specificity of the SL2-1 lectin for the core α1-6 fucose epitope on complex N-glycans. Additionally, these data support our observations with glycan array binding that SL2-1 has little or no affinity for a Fucα1-6GlcNAc disaccharide that remains after EndoS treatment of IgG. Finally, core fucosylation could easily be detected on nanogram quantities of glycoprotein using lectin blotting with biotinylated SL2-1 (Fig. 4b).

### Lectin-based ELISA for detection of core fucose on N-glycans

SL2-1 was tested in a direct lectin ELISA assay to determine its suitability to quantify core fucosylation of N-glycans on IgG. Titrated mouse monoclonal or rabbit polyclonal IgG was immobilized on a 96-well plate, then blocked with BSA and probed with DyLight 488-labeled SL2-1. Both mouse monoclonal IgG2a (anti-MBP, described above) and rabbit polyclonal IgG were efficiently detected (Fig. 4c). No signal was obtained in control samples treated with PNGase F, indicating the signal observed in this assay was specifically due to the presence of IgG N-glycans (data not shown). The difference in signal intensity between the two substrates correlated with prior observations that mouse and rabbit IgG N-glycans contain ~97% and <30% α1-6 fucose, respectively^21^. The standard curves for both IgG ELISA assays showed high correlation values. The assay was able to measure N-glycan core fucosylation of IgG from 312 to 2500 ng/mL with good linearity.

In a second ELISA assay, biotinylated SL2-1 was used with an HRP conjugated biotin antibody and a TMB colorimetric substrate. This assay detected IgG N-glycan core fucosylation of mouse IgG and rabbit IgG over a concentration range of 30 to 1000 ng/mL and 125 to 1000 ng/mL, respectively (Fig. 4d).

### SL2-1 mediated capture of core-fucosylated N-glycans from glycan mixtures

**Figure 5:**
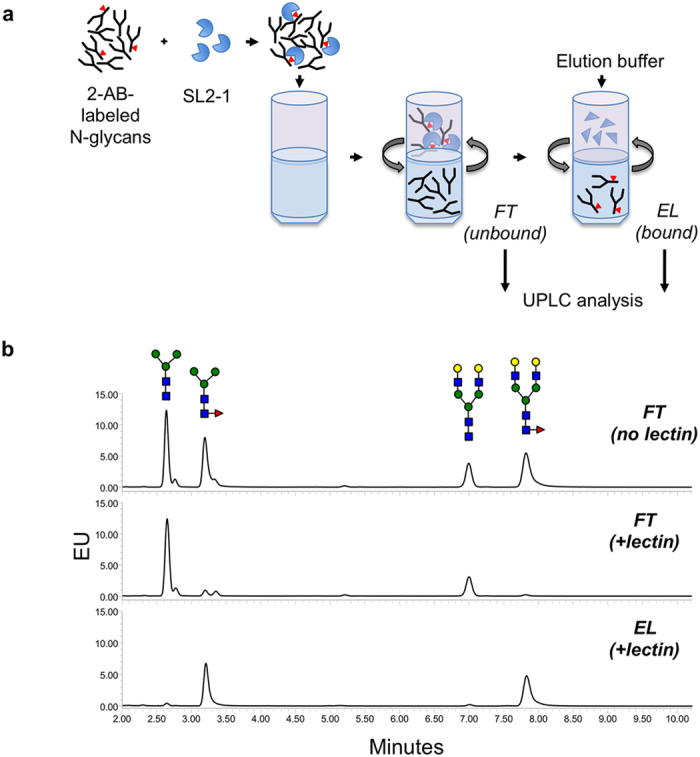
Lectin-based enrichment of core-fucosylated N-glycans. (**a**) A schematic of separation of lectin-captured N-glycans from unbound carbohydrates using centrifugal filter devices. (**b**) Enrichment of core-fucosylated N-glycans from a defined mixture of 2-AB-labeled glycan standards. Tri-mannosyl core and galactosylated biantennary glycans with or without fucose were pre-mixed and incubated in the presence or absence of SL2-1 before separation in the filter devices. N-Glycans from the flow-through and elution fractions were separately collected and analyzed by UPLC. In the absence of lectin, all the loaded N-glycans were recovered in the flow-through (FT) fraction (upper panel); unbound N-glycans (afucosylated) after incubation with SL2-1 were detected in the FT fraction (middle panel); lectin-enriched N-glycans (core fucosylated) were recovered in the elution fraction (EL) after proteolytic digestion of SL2-1 (lower panel).

SL2-1 was also used for profiling of fluorescently labeled core-fucosylated N-glycans using ultra-performance hydrophilic interaction liquid chromatography with fluorescence detection (UPLC-HILIC-FLR). A filter-aided sample preparation (FASP) method was employed for separation of lectin-bound N-glycans (core-fucosylated) from unbound N-glycans (afucosylated). For initial proof-of-principle experiments a mixture of four purified 2-aminobenzamide (2-AB)-labeled N-glycan standards was used: trimannosyl core N-glycan (paucimannose) with and without core α1-6 fucose (M3N2F and M3N2, respectively), and asialo-, galactosylated biantennary N-glycan with and without core α1-6 fucose (NA2F and NA2, respectively). A mixture of M3N2F, M3N2, NA2F and NA2 (4 pmol of each) was incubated with or without SL2-1, and unbound N-glycans were separated from lectin-bound glycans by centrifugation in Microcon-30 centrifugal filters (Fig. 5a). Collected flow-through (FT) fractions were analyzed by UPLC-HILIC-FLR. In the absence of the lectin, all four glycans were quantitatively recovered in the unbound FT fraction (Fig. 5b, upper panel). In the presence of lectin, 97–99% of non-fucosylated M3N2 and NA2 were recovered in the unbound FT fraction, whereas only 8% of M3N2F and 3% NA2F were detected in the flow-through fraction (Fig. 5b, middle panel), indicating that 92–97% of the fucosylated structures remained bound to SL2-1.

To release captured oligosaccharides from the SL2-1, several elution strategies were tested. No lectin-bound oligosaccharides were eluted after treatment with 0.3 M fucose, and only partial recovery (up to 25%) was achieved by elution with 0.2 M ammonium hydroxide or 5% formic acid. Proteolysis of SL2-1 by digestion with Proteinase K provided the most efficient and quantitative recovery of the captured glycans (95–98% recovery). Using this elution strategy, 91% of total loaded M3N2F and 95% of total loaded NA2F were recovered from the lectin-bound fraction (Fig. 5b, lower panel). These data indicate that SL2-1 binds N-glycans in complex samples in a highly selective manner, and permits efficient and quantitative isolation of core fucosylated N-glycans.

**Figure 6:**
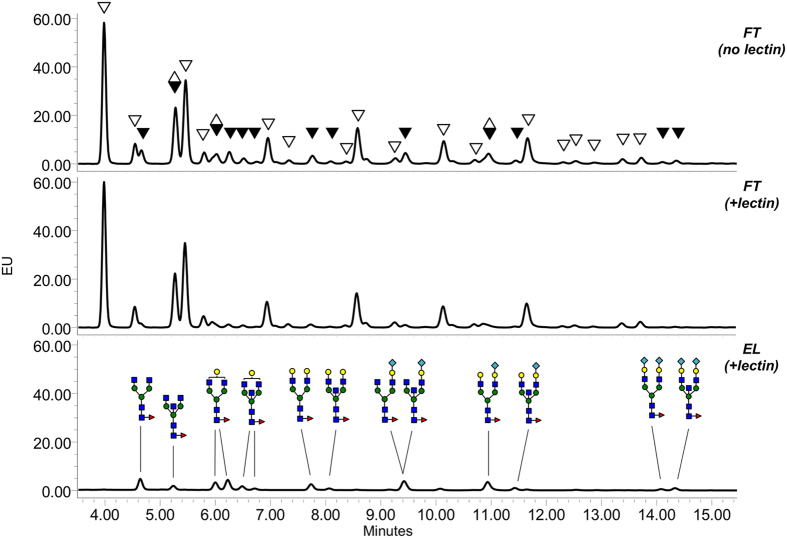
Lectin-based profiling of core-fucosylated N-glycans of rabbit serum IgG. Mixtures of 2-AB-labeled N-glycans from rabbit IgG were incubated in the presence or absence of SL2-1, and the lectin-bound and unbound fractions were separated and analyzed by UPLC. Black triangles indicate the peaks containing core-fucosylated N-glycans; open triangles indicate the peaks containing afucosylated N-glycans; diamonds indicate the peaks containing both core-fucosylated and afucosylated N-glycans. Peak annotation of the chromatograms was conducted using information obtained by LC-MS analysis and exoglycosidase treatment of the N-glycans.

This method was further tested by profiling of core fucosylated N-glycans from highly complex glycan mixtures. *N*-glycans from rabbit or human serum IgG were released by PNGase F and labeled with 2-AB. After incubation of the 2-AB-labeled glycans released from 15 μg of IgG (~200 pmol of N-glycans) with 30 μg of SL2-1, the fractions containing unbound and lectin-bound carbohydrates were collected and analyzed by UPLC-HILIC-FLR (as described above and in Materials and Methods). Profiles of the rabbit IgG samples showed that the FT fraction was effectively depleted of core fucosylated N-glycans, which were subsequently recovered in the elution fraction after proteolysis of SL2-1 (Fig. 6). The lectin-eluted N-glycans were further treated with α1-2,4,6 fucosidase (bovine kidney fucosidase, BKF), and all were BKF-sensitive (see Supplementary Fig. S5). Additionally, the profiles of N-glycans in the FT (unbound fraction) before and after BKF treatment were nearly identical indicating very efficient removal of core fucosylated N-glycans from the sample by SL2-1 (see Supplementary Fig. S5). The structures of both bound and unbound N-glycans were confirmed using LC-MS (see Supplementary Fig. S6) and the assigned structures of the lectin-bound glycans in the EL fraction are shown in Fig. 6. After completing peak annotation, N-glycan profiles of all fractions (including BKF-treated) were integratedusing Empower 3 software, and the relative area of each core-fucosylated glycan peak in FT (+lectin) and EL (+lectin) was compared to its corresponding peak area prior to lectin binding (FT (no lectin)). SL2-1 captured core fucosylated N-glycans with 91–98% efficiency (see Supplementary Table S3).

**Figure 7:**
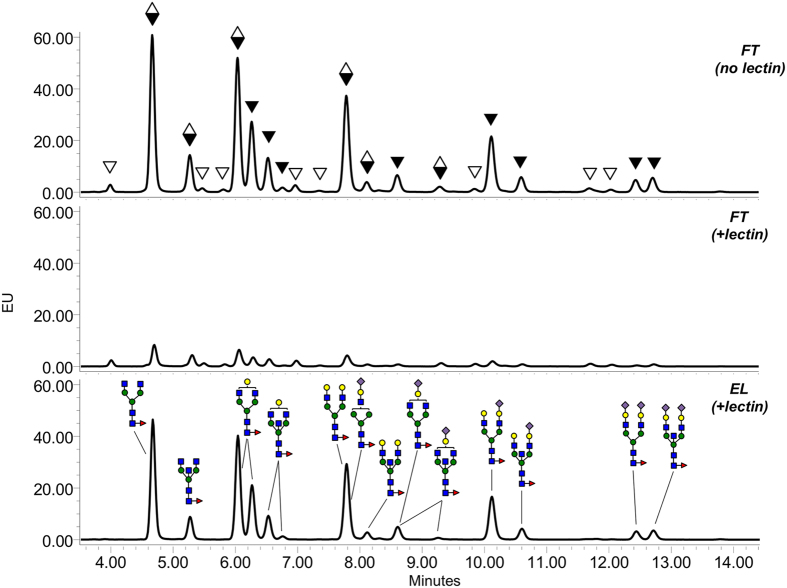
Lectin-based profiling of core-fucosylated N-glycans of human serum IgG. Mixtures of 2-AB-labeled N-glycans of human IgG were incubated with or without SL2-1, and the lectin-bound and unbound fractions were separated and analyzed by UPLC. Black triangles indicate the peaks containing core-fucosylated N-glycans; open triangles indicate the peaks containing afucosylated N-glycans; diamonds indicate the peaks containing both core-fucosylated and afucosylated N-glycans. Peak annotation of the chromatograms was conducted using information obtained by LC-MS analysis, exoglycosidase treatment of the N-glycans and comparison to the published data^22^.

A second analysis was performed with human IgG N-glycans using the same enrichment protocol. In contrast to rabbit IgG N-glycans that possess few core fucosylated N-glycans, most N-glycans from human IgG are core-fucosylated^23^. SL2-1 effectively and specifically isolated core α1-6 fucosylated N-glycans released from human IgG (Fig. 7). The isolated structures were confirmed by both BKF digestion (see Supplementary Fig. S7) and LC-MS analysis (see Supplementary Fig. S8). The assessed lectin capture efficiency of individual core fucosylated N-glycans from the human IgG N-glycan mixture was 87–97% (see Supplementary Table S4).

In summary, these data illustrate that SL2-1 can specifically and quantitatively isolate core fucosylated N-glycans from complex N-glycans mixtures. This method can be used to profile core fucosylated N-glycans from a broad range of biological samples or to subtract core fucosylated N-glycans from a sample to permit profiling and characterization of non-core fucosylated N-glycans. Furthermore, the method provides reliable relative quantitation of core fucosylated N-glycans.

## Discussion

In the present study, we identified and characterized the specificity of the fucose binding lectin SL2-1 from the bacterium *Streptomyces rapamycinicus*. SL2-1 was highly expressed in *E. coli* and purified recombinant SL2-1 was subjected to comprehensive glycan binding analyses. Our studies show that SL2-1 was highly specific for fucosylated chitobiose, an epitope found on many mammalian N-glycans. We further demonstrated that recombinant SL2-1 could be used in several analytical applications aimed at detecting and/or quantifying the presence of core fucosylation of N-glycans. Finally, we used SL2-1 in a new method for epitope-mediated profiling of core fucosylated N-glycans in complex mixtures using UPLC-HILIC-FLR.

SL2-1 belongs to a group of hypothetical bacterial and fungal proteins that exhibit similar domain organization and primary sequence similarity. SL2-1 is a single-chain polypeptide (~17 kDa) comprised of three short and highly similar repeating domains. Each individual repeat shows homology to the previously identified fungal fucose binding lectins RSL and PhoSL (73–76% and 82–84%, respectively). However, these fungal lectins were originally isolated directly from *Rhizopus stolonifer* and *Pholiota squarrosa* by purification of very small (~4.5 kDa) fucose binding peptides whose sequences were determined by protein sequencing techniques^12^,^14^. Thus, the native genomic sequences encoding these two peptides were not previously identified.

In our bioinformatics searches, we identified two highly similar protein sequences from basidiomycete fungi, in addition to several bacterial protein sequences of unknown function. One of the fungal sequences was from *Pholiota nameko*, a close relative of *Pholiota squarrosa* from which PhoSL derives. Both new fungal protein sequences showed high primary sequence similarity and domain organization to SL2-1, consisting of a single open reading frame with a predicted molecular weight of 16–17 kDa, and comprised of three tandem repeats of the PhoSL-like peptide. This three-domain architecture was also conserved in all of the bacterial sequences we identified in this study. Finally, the domain organization of SL2-1 also resembles another small actinomycete lectin, actinohivin, which specifically binds to high-mannose type saccharide chains^24^,^25^. Like SL2-1, actinohivin consists of three highly conserved repeating segments. These segments assemble in a similar manner to the beta-trefoil fold of ricin toxin and other carbohydrate-binding module family 13 proteins. Together, these observations support the notion that members of SL2-1 fucose binding lectin family are typically proteins having a triplicate repeat architecture. It is likely that the previously purified native RSL and PhoSL fucose binding peptides represent processed fragments of larger lectins consisting of three domains.

Several small lectins with the strict specificity for core α1,6 fucosylation of N-glycans have recently been identified in mushrooms and red algae (*i.e.*, PhoSL, hypninA, BTL)^12^,^13^,^14^,^15^. The observed binding affinities of SL2-1 for core fucosylated N-glycans (*K*_*a*_ of 6.3–9.45 × 10^5 ^M^−1^) were similar or better than those reported for PhoSL (*K*_*a*_ of 1.2–5 × 10^5 ^M^−1^), hypninA (*K*_*a*_ of 0.52–7.58 × 10^6 ^M^−1^) and BTL (*K*_*d*_ of 12 × 10^−6 ^M) lectins^13^,^14^,^15^. However, despite the general similarities in affinity and specificity of these lectins, there are differences that affect their broad utility as glycobiology tools. For example, PhoSL, BTL and hypninA were purified from native sources. Thus, a source of protein is not widely available for applied use. In contrast, both BTL and SL2-1 (this study) have been abundantly produced in recombinant form. However, recombinant BTL was less active than its native counterpart and was unstable upon lyophilization^15^. BTL and SL2-1 also slightly differ in their glycan binding specificities. BTL has some bias against binding core-fucosylated N-glycans that also possess bisecting GlcNAc^15^, potentially limiting its application for N-glycan enrichment and profiling strategies such as those demonstrated with SL2-1 in this study. We have shown that SL2-1 can efficiently enrich core-fucosylated N-glycans with or without bisecting GlcNAc from complex mammalian glycan mixtures (Figs 6 and 7).

Profiling N-glycans from mammalian samples often involves dealing with significant structural heterogeneity, which can be challenging to accurately interpret. For example, over 140 structural variants of *N*-glycans have been identified on glycoproteins present in human serum^26^. Approaches for dealing with N-glycan structural heterogeneity and improving the accuracy of the structural assignments that are made often involve multiple analytical techniques: the use of orthogonal analytical approaches (often liquid chromatography coupled to in-line or offline mass spectrometry), cross-referencing glycan mobilities and masses to databases of known structures, or exoglycosidase treatments to confirm the presence or absence of specific sugars in each glycan^27^.

In the present study, we exploited the highly precise binding specificity of SL2-1 to stratify complex mixtures of N-glycans by the presence or absence of the core fucose epitope. The presented method permits both structural profiling and relative quantification of core fucosylated glycans captured from different samples. This method could in principle be extended to other highly specific lectins that recognize other important glycan epitopes (*e.g*., Neu5Ac, Neu5Gc, terminal Gal, terminal GlcNAc, etc.). It is plausible that an array of highly precise lectins could be used in parallel or in series to reduce sample complexity while providing insight into certain sugar epitopes present on glycans in a profile. Such a method could assist in the analysis of highly complex N-glycan samples (*e.g*., serum or other body fluids) and would provide an additional orthogonal method to enhance glycan structure determination.

## Methods and Materials

### Materials

Chemical reagents and solvents were from Sigma-Aldrich (St.Louis, MO). Oligosaccharide standards (A1F, NA2, NA2F, M3N2, M3N2F, N2F) were purchased from ProZyme (Hayward, CA). Rapid Peptide-N-Glycosidase (PNGase F), Remove-iT^®^ Endo S, α1-2,4,6 Fucosidase, and Proteinase K were from New England Biolabs (Ipswich, MA). Rabbit and human serum IgG antibodies were obtained from Sigma-Aldrich.

### Cloning, expression and purification of SL2-1

A gene encoding hypothetical protein M271_34010 (GenBank accession: AGP58216.1) was found in the *Streptomyces rapamycinicus* NRRL 5491 genomic database^28^ (GenBank accession: CP006567.1) by BLASTP using the amino acid sequences of PhoSL (*Pholiota squarrosa*) or RSL (*Rhizopus stolonifer*) (UniProtKB/Swiss-Prot accession: P83973.1) as queries. *De novo* synthesis of a codon optimized sequence encoding the putative *S. rapamycinicus* fucose-binding lectin without signal peptide (26-183 a.a.) was performed by Integrated DNA Technologies (Coralville, IA). To generate a construct for expression in *E. coli*, the synthesized DNA fragment was digested with NdeI and EcoRI, and inserted into the corresponding sites of pET21a. The resulting plasmid was designated pET21a/SL2-1.

For protein expression, an overnight culture of *E. coli* cells carrying pET21a/SL2-1 was diluted 1:100 in 2 L of LB medium supplemented with 100 μg/mL ampicillin and grown to 0.6 OD_600_ at 30 °C. The expression of recombinant protein was induced by addition of isopropyl-β-thiogalactopyranoside (IPTG) to a final concentration of 0.4 mM and shaking for 4 h at 30 °C. The cells were harvested by centrifugation and suspended in 50 mL of 20 mM sodium phosphate, pH 7.4, containing 150 mM NaCl. The cells were lysed by sonication with eight 20 s bursts. Cell debris was removed by centrifugation at 19,000 × *g* for 60 min at 4 °C. Recombinant protein SL2-1 was purified by affinity chromatography on 4 mL column packed with L-fucose agarose. Bound protein was eluted in five fractions (4 mL each) of elution buffer (20 mM sodium phosphate, pH 7.4 containing 150 mM NaCl and 300 mM L-fucose). Pooled elution fractions containing pure protein were dialyzed against 20 mM sodium phosphate, pH 7.4 and concentrated using Vivaspin 20 10,000 Da MWCO concentrators (Sartorius Stedim Biotech). Purified protein was stored at −20 °C.

### Biotinylation of SL2-1

SL2-1 was biotinylated using EZ-Link Sulfo-NHS-LC-Biotin (Thermo Scientific, Waltham, MA) according to the manufacturer’s recommendations. Briefly, 1.53 μmol of EZ-Link Sulfo-NHS-LC-Biotin (dissolved in water) added to 1 mL of SL2-1 (1.3 mg/mL, in 20 mM sodium phosphate, pH 7.4). After incubation for 60 min at room temperature, the mixture was dialyzed against 20 mM sodium phosphate, pH 7.4, 150 mM NaCl using a 7000 Da MWCO dialysis membrane and stored at −20 °C until used.

### Microtiter plate glycan binding assay

A Pierce™ streptavidin coated high capacity 96-well plate (Thermo Fisher Scientific) was coated by addition of 100 μL of biotinylated SL2-1 (15 μg/mL in phosphate-buffered saline [PBS]) to each well, followed by incubation for 4 hours at 25 °C. Uncoupled lectin was removed by washing the plate three times with PBS. Three nanomoles of 7-amino-4-methylcoumarin (AMC)-labeled glycan (in 100 μL of PBS) was added to each well, and incubated for 2 hours at 25 °C, after which the plate was washed 4 times with PBS, and the fluorescence intensity was measured at 340/440 nm (Ex/Em).

### Lectin ELISA

Nunc MaxiSorp flat-bottom 96-well plates (Thermo Fisher Scientific) were coated by addition of 100 μL of diluted glycoprotein in PBS to each well, and incubation overnight at 4 °C. The plates were then washed with PBST (PBS containing 0.05% Tween 20, pH 7.4) and blocked by addition of 200 μL PBS containing 1% bovine serum albumin (BSA) to each well, and incubation for 1 h at room temperature. Plates were washed 4 times with PBST, after which 100 μL of biotinylated lectin (5 μg/mL in PBS) was added to each well and incubated for 1 h at room temperature. The plates were then washed 4 times with PBST, followed by the addition of 100 μL of horseradish peroxidase-conjugated anti-biotin antibodies (Cell Signaling Technologies, Beverly, MA) to each well and incubation for 1 h at room temperature. The plates were again washed 4 times with PBST, and the 3, 3′, 5, 5′-tetramethylbenzidine (TMB) liquid substrate system (Sigma) was used for colorimetric detection of lectin-glycoprotein complexes. The detection reaction was stopped by addition of 100 μL of 1 M HCl, and the absorbance was measured at 450 nm. Black 96-well plates with clear bottom (Costar) were used for ELISA experiments with fluorescent dye-labeled lectin. The working concentration of DyLight 488 labeled SL2-1 in PBS was 0.9 μg/mL.

### Lectin western blotting

Different proteins (typically, 1–3 μg per load) were subjected to 10–20% SDS-PAGE. After electrophoresis, separated proteins were blotted onto nitrocellulose membranes. The membranes were blocked for 1 h with 3% dry milk in PBST, then for 1 h with PBST containing 0.6 μg/mL biotinylated SL2-1 and 3% dry milk. After incubation with the SL2-1, the membranes were washed with PBST four times for 5 min, and incubated 1 h with horseradish peroxidase-conjugated anti-biotin antibodies (Cell Signaling Technologies, Beverly, MA). The membranes then were washed with PBST, and targets visualized using SuperSignal West Pico chemiluminescent substrate (Thermo Scientific).

### Isothermal titration calorimetry measurements

Purified SL2-1 was extensively dialyzed against 20 mM sodium phosphate, pH 7.4 and degassed. Carbohydrate ligands were dissolved in the same buffer, degassed, and loaded in the 50 μL injection syringe. Isothermal titration calorimetry (ITC) was performed using a Nano ITC microcalorimeter (TA Instruments). The SL2-1 sample (19 μM final concentration) was loaded into a 190 μL sample cell at 25 °C. Titration was performed with 25 injections of 1.84 μL carbohydrate ligand every 300 s with a stirring at 300 rpm. Data were fitted with NanoAnalyze^TM^ software according to standard procedures. The fitted data yielded the stoichiometry (*n*), association constant (*K*_*a*_), dissociation constant (*K*_*d*_) and enthalpy of binding (Δ*H*). Other thermodynamic parameters (*i.e.* changes in free energy (Δ*G*) and entropy (Δ*S*)) were calculated from the equation Δ*G* = Δ*H *− *T*Δ*S* = −*RT*ln*K*_*a*_, in which *T* is the absolute temperature and *R* = 8.314 J mol^−1^ *K*^−1^. Two independent titrations were performed for each ligand tested.

### Mammalian glycan array screening

Purified SL2-1 was labeled with DyLight 488 NHS Ester (Thermo Scientific) according to the manufacturer’s instructions and dialyzed against 20 mM Tris-HCl, pH 7.4, 150 mM NaCl using 10,000 MWCO dialysis membrane. DyLight 488 labeled protein was used for glycan array screening at the Protein-Glycan Interaction Core H of the Consortium for Functional Glycomics (Emory University, Atlanta, GA).

The specificity of SL2-1 was determined by screening its binding on the printed array (version 5.2) consisting of 609 mammalian glycans. Detailed information about the structures and linkers of these glycans can be found at https://glycopattern.emory.edu. The printed array was probed with 200 μg/mL of SL2-1 diluted in 20 mM Tris-HCL pH 7.4, 150 mM NaCl, 2 mM CaCl_2_, 2 mM MgCl_2_, 0.05% Tween 20, 1% BSA, in 6 replicate binding experiments. The highest and lowest point from each set of six replicates was discarded, and the average RFU value of 4 replicates, the standard deviation, and % CV (% CV = 100 * Standard Deviation/Mean) was calculated.

### Deglycosylation of antibodies and cleanup of liberated glycans

Aliquots (150 μg) of mouse monoclonal anti-MBP antibody (New England Biolabs), human or rabbit polyclonal IgG (Sigma-Aldrich) were incubated with Rapid PNGase F (New England Biolabs) in a total volume of 50 μL of Rapid PNGase F reaction buffer for 10 min at 50 °C. For use in lectin ELISA experiments, the deglycosylated antibodies were dialyzed against 20 mM sodium phosphate, pH 7.4, 150 mM NaCl using 10,000 Da MWCO dialysis membranes. For glycan labeling, the liberated N-glycans were cleaned and desalted using SPE Supelclean columns ENVI-18 100 mg and ENVI-CARB 100 mg (Supelco).

### 2-AB labeling and cleanup of excess label

Fluorescent labeling mix (10 μL; 350 mM 2-aminobenzamide (2-AB), 1 M sodium cyanoborohydride in acetic acid/dimethyl sulfoxide (30:70)) was added into each tube containing dried N-glycan sample, and the tube was incubated at 65 °C with agitation at 700 rpm for 120 min. Excess label was removed by HILIC SPE using MacroSpin columns (Nest Group, Inc.).

### Lectin capture of core-fucosylated N-glycans

2-AB labeled N-glycan standards (4 pmol; Prozyme) were incubated with 20 μg of SL2-1 in 100 μL of 10 mM Tris-HCl, pH 8.0 for 1 h at room temperature. Binding reaction mixes were transferred to Microcon-30 (Ultracel YM-30, Merck Millipore) centrifugal concentration devices that had been washed with 500 μL of deionized water. The concentrators were centrifuged for 5 min at 11,000 × *g*, and the filtrates collected. The filters were washed twice with 120 μL of 10 mM Tris-HCl, pH 8.0 buffer, centrifuged as above, and the washes combined with the initial filtrate. The pooled fractions contain N-glycans that did not bind SL2-1 and flowed through the device (FT fraction). To elute N-glycans that had bound to SL2-1, the filter devices were transferred to new collection tubes and 130 μL proteinase K mix (6 units of proteinase K without glycerol in 10 mM Tris-HCl, pH 8.0) was added to each device. The reaction was incubated at 42 °C for 4–6 hours. The concentrators were again centrifuged for 5 min, washed twice, and filtrates pooled as described above. The pooled fractions contain eluted N-glycans that bound to SL2-1 (EL fraction). The FT and EL fractions were dried using SpeedVac concentrator and each sample dissolved in 10 μL of deionized water. For UPLC-HILIC-FLR analysis, 5 μL of each sample was mixed with 11.7 μl acetonitrile (final ratio 30:70 water/acetonitrile). A 5-10 μL aliquot of this mix was used for UPLC-HILIC-FLR separation.

### UPLC-HILIC-FLR

2-AB-labeled N-glycans were separated by UPLC using a Waters Acquity BEH glycan amide column (2.1 × 150 mm, 1.7 μm) on a Waters H-Class ACQUITY instrument (Waters Corporation) equipped with a quaternary solvent manager and a fluorescence detector. Solvent A was 50 mM ammonium formate buffer pH 4.4 and solvent B was acetonitrile. The gradient used was 0–1.47 min, 30% solvent A; 1.47–24.81 min, 30–47% solvent A; 25.5–26.25 min, 70% solvent A; 26.55–32 min, 30% solvent A. The flow rate was 0.561 mL/min. The injection volume was 10 μL and the sample was prepared in 70% (v/v) acetonitrile. Samples were kept at 5 °C prior to injection and the separation temperature was 40 °C. The fluorescence detection wavelengths were λex = 330 nm and λem = 420 nm with a data collection rate of 20 Hz. A dextran hydrolysate ladder was used to convert retention times into glucose unit (GU) values. All data was processed using Waters Empower 3 chromatography workstation software.

## Additional Information

**How to cite this article**: Vainauskas, S. *et al*. Profiling of core fucosylated N-glycans using a novel bacterial lectin that specifically recognizes α1,6 fucosylated chitobiose. *Sci. Rep.* **6**, 34195; doi: 10.1038/srep34195 (2016).

## Supplementary Material

Supplementary Information

## Figures and Tables

**Table 1:** SL2-1 interaction with different fucosylated ligands measured by isothermal titration calorimetry (ITC).

	*K*_*a*_	*K*_*d*_	*n*	*ΔG*	*ΔH*	*TΔS*
	*(M*^*−1*^)	*(M)*		*(kJ/mol)*	*(kJ/mol)*	*(kJ/mol)*
N2F*	6.3 × 10^5^	1.59 × 10^−6^	2.01	−33.11	−46.36	−13.25
A1F**	9.45 × 10^5^	1.06 × 10^−6^	1.85	−34.12	−49.18	−15.06

^*^GlcNAcβ1-4(Fucα1-6)GlcNAc.

^**^Neu5Acα2-6Galβ1-4GlcNAcβ1-2Manα1-6(Galβ1-4GlcNAcβ1-2Manα1-3)Manβ1-4GlcNAcβ1-4(Fucα1-6)GlcNAc.
