# Supplementary material for: Profiling of core fucosylated N-glycans using a novel bacterial lectin that specifically recognizes α1,6 fucosylated chitobiose
Source: Sci Rep. 2016 Sep 28;6:34195. doi: 10.1038/srep34195 (PMC5039751; doi:10.1038/srep34195)
Supplement: Supplementary Information [file srep34195-s1.pdf]

Supplementary Information

Profiling of core fucosylated N-glycans using a novel bacterial lectin that specifically recognizes α1,6 fucosylated chitobiose

Saulius Vainauskas[1], Rebecca M. Duke[1,2], James McFarland[1], Colleen McClung[1], Cristian Ruse[1] and Christopher H. Taron[1,*]

[1] New England Biolabs, 240 County Road, Ipswich, MA 01938, USA

[2] Charles River Laboratories, 8 Henshaw St., Woburn, MA 01801, USA

* Corresponding author. Email: taron@neb.com

This document provides information supplemental to the main text, in the following sections:

A - Supporting Experimental Section

B - Supporting Tables and Figures

A - Supporting Experimental Section

Gel Filtration

Gel filtration for estimation of the molecular weight was performed using a HiPrep 16/60 Sephacryl S-200 HR column (GE Healthcare Life Sciences) equilibrated with 20 mM sodium phosphate, pH 7.4 containing 150 mM NaCl. Elution was carried out with the same buffer at a flow rate of 0.5 ml/min. The calibration proteins were used as follows: conalbumin (75 kDa), ovalbumin (43 kDa), carbonic anhydrase (29 kDa), ribonuclease A (13.7 kDa), and aprotinin (6.5 kDa).

Liquid chromatography mass spectrometry analysis (LC-MS)

The lectin sample was diluted in 0.1% formic acid/water and analyzed by reverse phase liquid chromatography (LC) and electrospray ionization time-of-flight mass spectrometry (ESI-TOF MS). A custom reverse phase chip (Agilent Technologies), containing an integrated trapping column (40 nl capacity), separation column and nano-ESI emitter (75 mm x 150 mm both packed with PLRP-S, 5 mm particles, 1000 A pore size) was used for the separation of proteins. The chip trapping column was loaded at 4 ml/min and the separation column developed at a flow rate of 600 nl/min using an Agilent 1200 series nano-LC coupled to an Agilent 6210 series ESI-TOF mass spectrometer. The column was equilibrated with 0.1% formic acid water containing 5% acetonitrile. 140 ng of protein was injected onto the column and it was developed after two minutes with a twenty-five minute linear gradient from 5% to 95% acetonitrile. Protein was found to elute at approximately twelve minutes post-injection. The mass spectra were acquired from 100-3200 m/z, one cycle/sec and 10,000 transients per scan using an ionization energy of 1800 V, fragmentor of 215 V, and drying gas of 275 C at 4.0 l/min. The acquired spectra were extracted and the protein spectra deconvoluted with Agilent MassHunter Qualitative Analysis Software (with Bioconfirm) B 2.0.2 software using a mass range of 10k to 100k Daltons.

UPLC-HILIC-FLR and UPLC-HILIC-FLR-MS

The 2-AB-labeled N-glycans were separated by UPLC using a Waters ACQUITY BEH Glycan Amide column (2.1 x 150 mm, 1.7 μm) that was installed on a Waters H-Class ACQUITY instrument equipped with a quatenary solvent manager and a fluorescence detector. Solvent A was 50 mM ammonium formate buffer pH 4.4 and solvent B was acetonitrile. The gradient used was 0-1.47 min, 30% solvent A; 1.47-24.81 min, 30–47% solvent A; 25.5-26.25 min, 70% solvent A; 26.55-32 min, 30% solvent A. The flow

rate was 0.561 mL/min. The injection volume was 10 µL and the sample was prepared in 70% (v/v) acetonitrile. Samples were kept at 5°C prior to injection and the separation temperature was 40°C. The fluorescence detection wavelengths were λex = 330 nm and λem = 420 nm with a data collection rate of 20 Hz. A dextran hydrolysate ladder was used to convert retention times into glucose unit (GU) values. All data was processed using Waters Empower 3 chromatography workstation software.

HPLC-HILIC-FLR-MS was performed using a Waters XBridge™ BEH Amide coloumn (3 × 150 mm, 2.5 um) on a Dionex UltiMate® LC, in line with a LTQ™ Velos™ Pro Mass Spectrometer equiped with a heated electrospray standard source (HESI-II probe). The flow rate was 0.300 mL/min and the column temperature was maintained at 30°C; solvent A was 50 mM ammonium formate buffer pH 4.4 and solvent B was acetonitrile. A linear gradient of 30% to 47% A over 50 min was used. The injection volume was 98 µL and the sample was prepared in 80% (v/v) acetonitrile. MS data was acquired in positive ion mode with a source voltage of 3 kV and capillary temperature of 275°C. Sheath gas flow, Aux gas and sweep gas flow rates were 11, 5 and 0, respectively. Full scan data for rabbit and human N-glycans were aquired over a m/z range of 400 to 2000. All data was processed using Thermo™ Xcalibur Roadmap.

BKF treatment

Recombinant α(1-2/4/6)-fucosidase (New England Biolabs) was used for the structural characterization of the glycans. 2AB-labeled N-glycans were incubated with 32 mU of α(1-2/4/6)-fucosidase in 20 µL of 50 mM sodium acetate buffer (pH 5.5) for 24-48 h at 37°C. After digestion, the enzymes were removed from the sample by filtration through a 10 kDa MWCO ultrafiltration plate (Pall LifeSciences). The filtrate was dried in a SpeedVac and re-suspended in H_2O for analysis by UPLC-HILIC-FLR.

B – Supporting Tables and Figures

Table S1. Representation of structures of AMC-labeled oligosaccharides used for microtiter plate glycan binding screening.

Glycan	Structure*
1	GlcNAcβ1-2Manα1-6(GlcNAcβ1-4)(GlcNAcβ1-2Manα1-3)Manβ1-4GlcNAcβ1-4GlcNAc
2	Neu5Acα2-6GalNAc
3	Galβ1-4GlcNAc
4	GlcNAcβ1-4GlcNAcβ1-4GlcNAcβ1-4GlcNAc
5	GalNAcα1-3(Fucα1-2)Gal
6	Fucα1-2Galβ1-4Glc
7	Galβ1-3(Neu5Acα2-6)GlcNAcβ1-3Galβ1-4Glc
8	Fucα1-2Galβ1-3(Fucα1-4)GlcNAcβ1-3Galβ1-4Glc
9	Neu5Acα2-6Galβ1-4GlcNAcβ1-3Galβ1-4Glc
10	Galβ1-3GalNAcβ1-4(Neu5Acα2-3)Galβ1-4Glc
11	Neu5Acα2-8Neu5Acα2-8Neu5Acα2-8Neu5Ac
12	Glcα1-4Glcα1-4Glc
13	Galβ1-3(Fucα1-4)GlcNAcβ1-3Galβ1-4(Fucα1-3)GlcNAcβ1-3Galβ1-4Glc
14	Galβ1-4(Fucα1-3)GlcNAcβ1-6(Fucα1-2Galβ1-3(Fucα1-3)GlcNAcβ1-3)Galβ1-4Glc
15	Galα1-3Galβ1-4Galα1-3Gal
16	Galα1-3Gal
17	Manα1-6(Manα1-3)Manα1-6(Manα1-3)Man
18	GlcNAcβ1-2Manα1-6 (GlcNAcβ1-2Manα1-3)Man
19	Neu5Acα2-3Galβ1-3GlcNacβ1-3Galβ1-4Glc
20	Xyl
21	GlcAβ1-3GlcNAcβ1-4GlcAβ1-3GlcNAcβ1-4GlcAβ1-3GlcNAcβ1-4GlcAβ1-3GlaNAc
22	Xylβ1-4Xylβ1-4Xyl
23	Manβ1-4Manβ1-4Man
24	Xylβ1-4Xylβ1-4Xylβ1-4Xyl
25	Araα1-5Araα1-5Araα1-5Ara
26	Galβ1-3(Fucα1-4)GlcNAcβ1-3Glc
27	Galα1-6Galα1-6Glcα1-2Fru
28	Manα1-3Manβ1-4GlcNAc
29	Neu5Acα2-3,6 Galβ1-4GlcNAcβ1-2 Manα1-3 (Neu5Acα2-3,6Galβ1-4GlcNAcβ1-2 Manα1-3)Manβ1-4GlcNAcβ1-4GlcNAc
30	Manβ1-4Man
31	GalNAc
32	GalA
33	GlcA
34	Manα1-6(Manα1-3)Manβ1-4GlcNAcβ1-4(Fucα1-6)GlcNAc
35	GlcNAcβ1-4(Fucα1-6)GlcNAc
36	Galβ1-4GlcNAcβ1-2Manα1-6(GlcNAcβ1-Manα1-3)Manβ1-4GlcNAcβ1-4(Fucα1-6)GlcNAc
37	Galα1,4Galβ1-4Glc
38	Manα1-3Manβ1-4GlcNAc
39	Neu5Acα2-6Galβ1-4GlcNAcβ1-2(Neu5Acα2-3Galβ1-3 (Neu5Acα2-6)GlcNAcβ1-4)Manα1-3(Neu5Acα2-3,6Galβ1-4GlcNAcβ1-2 Manα1-6)Manβ1-4GlcNAcβ1-4GlcNAc
40	Glcβ1-4Glcβ1-4Glcβ1-4Glc
41	Neu5Acα2-3,6 Galβ1-3GalNAc

42	GalNAcα1-3(Fucα1-2)Galβ1-4Glc
43	Manα1-6(Manα1-3)Manα1-6(Manα1-3)Manβ1-4GlcNAc
44	Galβ1-4Glc
45	Galβ1-4(Fucα1-3)GlcNAcβ1-3Galβ1-4Glc
46	Galα1-3(Fucα1-2)Galβ1-4(Fucα1-2)Glc
47	Galβ1-4GlcNAcβ1-6(Galβ1-4GlcNAcβ1-2)Manα1-6Galβ1-4GlcNAcβ1-4(Gal β1-4GlcNAcβ1-2)Manα1-3)Manβ1-4GlcNAcβ1-4GlcNAc
48	GlcNAcβ1-4GlcNAcβ1-4GlcNAc
49	Galβ1,4(Fucα1-3)GlcNAcβ1-6(Galβ1-3(Fucα1-4)GlcNAc b1,3)Galβ1-4Glc
50	Galβ1-4(Fucα1-3)GlcNAcβ1-6(Fucα1-2Galβ1-3(Fucα1-4)GlcNAcβ1-3)Galβ1-4Glc
51	Galβ1-4(Fucα1-3)GlcNAcβ1-3Galβ1-4-Glc
52	GalNAcα1-3(Fucα1-2)Galβ1-4Glc
53	Manα1-6Manα1-6Man
54	Manα1-6(Manα1-3)Manβ1-4GlcNAcβ1-4GlcNAc
55	Manα1-6(Manα1-3)Manα1-6(Manα1-3)Manβ1-4GlcNAcβ1-4GlcNAc
56	Fucα1-6GlcNAc

* GlcNAc, N-Acetyl-D-glucosamine; Man, D-Mannose; Neu5Ac, N-Acetylneuraminic acid; GalNAc, N-Acetyl-D-galactosamine; Gal, D-Galactose; Fuc, D-Fucose; Glc, D-Glucose; Xyl, D-Xylose; Ara, D-Arabinose; GalA, D-Galactouronic acid; GlcA, D-Glucuronic acid

Table S2. Mammalian glycan array (CFG) data for SL2-1. Only results of the core-fucosylated N-glycans are presented in the table. The data is arranged by RFU (high to low) to provide a list of the glycans bound with highest intensity. The rank number corresponds to the overall rank # in the complete list of 609 glycans. RFU values for available analogous of non-core-fucosylated glycans are also provided.

Rank	Glycan Structure (text)*	Glycan Structure (symbol)**	RFU	St Dev	% CV	Non-CF analog (RFU)
1	Galb1-4GlcNAcb1-3Galb1-4GlcNAcb1-3Galb1-4GlcNAcb1-3Galb1-4GlcNAcb1-3Galb1-4GlcNAcb1-6(Galb1-4GlcNAcb1-3Galb1-4GlcNAcb1-3Galb1-4GlcNAcb1-3Galb1-4GlcNAcb1-3Galb1-4GlcNAb1-2)Mana1-6(Galb1-4GlcNAcb1-3Galb1-4GlcNAcb1-3Galb1-4GlcNAcb1-3Galb1-4GlcNAcb1-3Galb1-4GlcNAcb1-2Mana1-3)Manb1-4GlcNAcb1-4(Fuca1-6)GlcNAcb-Sp24	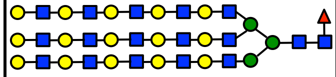	17143	215	1	n/a
2	GlcNAcb1-3Galb1-4GlcNAcb1-3Galb1-4GlcNAcb1-3Galb1-4GlcNAcb1-2Mana1-6(GlcNAcb1-3Galb1-4GlcNAcb1-3Galb1-4GlcNAcb1-3Galb1-4GlcNAcb1-2Mana1-3)Manb1-4GlcNAcb1-4(Fuca1-6)GlcNAcb-Sp24	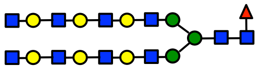	15311	352	2	431
3	GlcNAcb1-3Galb1-4GlcNAcb1-3Galb1-4GlcNAcb1-3Galb1-4GlcNAcb1-3Galb1-4GlcNAcb1-6(GlcNAcb1-3Galb1-4GlcNAcb1-3Galb1-4GlcNAcb1-3Galb1-4GlcNAcb1-3Galb1-4GlcNAb1-2)Mana1-6(GlcNAcb1-3Galb1-4GlcNAcb1-3Galb1-4GlcNAcb1-3Galb1-4GlcNAcb1-3Galb1-4GlcNAcb1-2Mana1-3)Manb1-4GlcNAcb1-4(Fuca1-6)GlcNAcb-Sp24	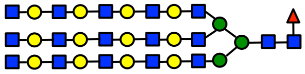	14766	777	5	n/a
4	Galb1-4GlcNAcb1-3Galb1-4GlcNAcb1-3Galb1-4GlcNAcb1-3Galb1-4GlcNAcb1-2Mana1-6(Galb1-4GlcNAcb1-3Galb1-4GlcNAcb1-3Galb1-4GlcNAcb1-3Galb1-4GlcNAcb1-2Mana1-3)Manb1-4GlcNAcb1-4(Fuca1-6)GlcNAcb-Sp24	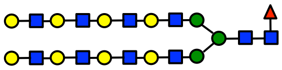	14336	1075	8	622
5	Galb1-4GlcNAcb1-6(Galb1-4GlcNAcb1-2)Mana1-6(Galb1-4GlcNAcb1-2Mana1-3)Manb1-4GlcNAcb1-4(Fuca1-6)GlcNAcb-Sp24	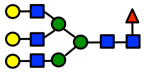	13565	844	6	399
6	Galb1-4GlcNAcb1-3Galb1-4GlcNAcb1-3Galb1-4GlcNAcb1-3Galb1-4GlcNAcb1-6(Galb1-4GlcNAcb1-3Galb1-4GlcNAcb1-3Galb1-4GlcNAcb1-3Galb1-4GlcNAb1-2)Mana1-6(Galb1-4GlcNAcb1-3Galb1-4GlcNAcb1-3Galb1-4GlcNAcb1-3Galb1-4GlcNAcb1-2Mana1-3)Manb1-4GlcNAcb1-4(Fuca1-6)GlcNAcb-Sp24	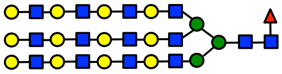	13209	987	7	n/a
7	GlcNAcb1-3Galb1-4GlcNAcb1-3Galb1-4GlcNAcb1-3Galb1-4GlcNAcb1-3Galb1-4GlcNAcb1-2Mana1-6(GlcNAcb1-3Galb1-4GlcNAcb1-3Galb1-4GlcNAcb1-3Galb1-4GlcNAcb1-3Galb1-4GlcNAcb1-2Mana1-3)Manb1-4GlcNAcb1-4(Fuca1-6)GlcNAcb-Sp19	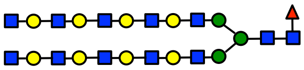	12875	1156	9	399
8	Fuca1-2Galb1-4(Fuca1-3)GlcNAcb1-2Mana1-6(Fuca1-2Galb1-4(Fuca1-3)GlcNAcb1-2Mana1-3)Manb1-4GlcNAcb1-4(Fuca1-6)GlcNAcb-Sp24	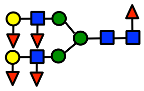	11738	1064	9	187
9	Neu5Aca2-6Galb1-4GlcNAcb1-2Mana1-6(Neu5Aca2-6Galb1-4GlcNAcb1-2Mana1-3)Manb1-4GlcNAcb1-4(Fuca1-6)GlcNAcb-Sp24	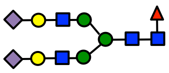	10358	779	8	51

10	Galb1-3GlcNAcb1-3Galb1-4GlcNAcb1-3Galb1-4GlcNAcb1-6(Galb1-3GlcNAcb1-3Galb1-4GlcNAcb1-3Galb1-4GlcNAb1-2)Mana1-6(Galb1-3GlcNAcb1-3Galb1-4GlcNAcb1-3Galb1-4GlcNAcb1-2Mana1-3)Manb1-4GlcNAcb1-4(Fuca1-6)GlcNAcb-Sp24	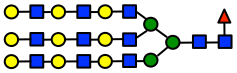	10241	166	2	n/a
11	GlcNAcb1-6(GlcNAcb1-2)Mana1-6(GlcNAcb1-2Mana1-3)Manb1-4GlcNAcb1-4(Fuca1-6)GlcNAcb-Sp24	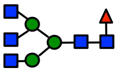	9832	538	2	52
12	Galb1-4GlcNAcb1-3Galb1-4GlcNAcb1-3Galb1-4GlcNAcb1-3Galb1-4GlcNAcb1-3Galb1-4GlcNAcb1-2Mana1-6(Galb1-4GlcNAcb1-3Galb1-4GlcNAcb1-3Galb1-4GlcNAcb1-3Galb1-4GlcNAcb1-3Galb1-4GlcNAcb1-2Mana1-3)Manb1-4GlcNAcb1-4(Fuca1-6)GlcNAcb-Sp19	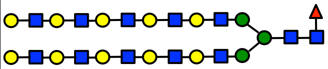	9711	1226	13	630
13	Galb1-4GlcNAcb1-3Galb1-4GlcNAcb1-2Mana1-6(Galb1-4GlcNAcb1-3Galb1-4GlcNAcb1-2Mana1-3)Manb1-4GlcNAcb1-4(Fuca1-6)GlcNAcb-Sp24	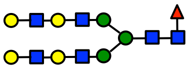	8211	1573	19	288
14	Galb1-4GlcNAcb1-3Galb1-4GlcNAcb1-3Galb1-4GlcNAcb1-6(Galb1-4GlcNAcb1-3Galb1-4GlcNAcb1-3Galb1-4GlcNAb1-2)Mana1-6(Galb1-4GlcNAcb1-3Galb1-4GlcNAcb1-3Galb1-4GlcNAcb1-2Mana1-3)Manb1-4GlcNAcb1-4(Fuca1-6)GlcNAcb-Sp24	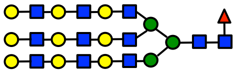	7999	1503	19	n/a
15	GlcNAcb1-3Galb1-4GlcNAcb1-3Galb1-4GlcNAcb1-2Mana1-6(GlcNAcb1-3Galb1-4GlcNAcb1-3Galb1-4GlcNAcb1-2Mana1-3)Manb1-4GlcNAcb1-4(Fuca1-6)GlcNAcb-Sp24	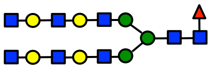	7646	527	7	145
16	GlcNAcb1-3Galb1-4GlcNAcb1-3Galb1-4GlcNAcb1-6(GlcNAcb1-3Galb1-4GlcNAcb1-3Galb1-4GlcNAb1-2)Mana1-6(GlcNAcb1-3Galb1-4GlcNAcb1-3Galb1-4GlcNAcb1-2Mana1-3)Manb1-4GlcNAcb1-4(Fuca1-6)GlcNAcb-Sp24	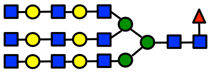	7045	797	11	n/a
17	Galb1-4GlcNAcb1-3Galb1-4GlcNAcb1-6(Galb1-4GlcNAcb1-3Galb1-4GlcNAb1-2)Mana1-6(Galb1-4GlcNAcb1-3Galb1-4GlcNAcb1-2Mana1-3)Manb1-4GlcNAcb1-4(Fuca1-6)GlcNAcb-Sp24	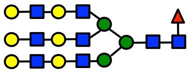	6845	430	6	779
18	GlcNAcb1-3Galb1-4GlcNAcb1-3Galb1-4GlcNAcb1-3Galb1-4GlcNAcb1-6(GlcNAcb1-3Galb1-4GlcNAcb1-3Galb1-4GlcNAcb1-3Galb1-4GlcNAb1-2)Mana1-6(GlcNAcb1-3Galb1-4GlcNAcb1-3Galb1-4GlcNAcb1-3Galb1-4GlcNAcb1-2Mana1-3)Manb1-4GlcNAcb1-4(Fuca1-6)GlcNAcb-Sp24	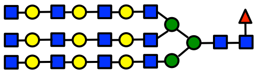	6100	815	13	n/a
19	Galb1-4GlcNAcb1-3Galb1-4GlcNAcb1-3Galb1-4GlcNAcb1-2Mana1-6(Galb1-4GlcNAcb1-3Galb1-4GlcNAcb1-3Galb1-4GlcNAcb1-2Mana1-3)Manb1-4GlcNAcb1-4(Fuca1-6)GlcNAcb-Sp24	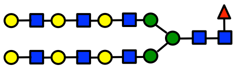	5959	970	16	21
20	Galb1-4GlcNAcb1-6(Galb1-4GlcNAcb1-2)Mana1-6(GlcNAcb1-4)Galb1-4GlcNAcb1-4(Gal b1-4GlcNAcb1-2)Mana1-3)Manb1-4GlcNAcb1-4(Fuca1-6)GlcNAc-Sp21	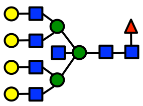	5929	1156	19	18
21	GlcNAcb1-3Galb1-4GlcNAcb1-2Mana1-6(GlcNAcb1-3Galb1-4GlcNAcb1-2Mana1-3)Manb1-4GlcNAcb1-4(Fuca1-6)GlcNAcb-Sp24	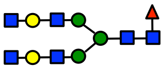	5659	352	6	417

22	GlcNAcb1-2 Mana1-6(GlcNAcb1-4)(GlcNAcb1-2Mana1-3)Manb1-4GlcNAcb1-4(Fuca1-6)GlcNAc-Sp21	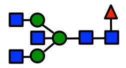	5610	377	7	14
23	Galb1-4GlcNAcb1-2 Mana1-6(Galb1-4GlcNAcb1-4)(Galb1-4GlcNAcb1-2Mana1-3)Manb1-4GlcNAcb1-4(Fuca1-6)GlcNAc-Sp21	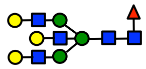	5093	636	12	n/a
24	Neu5Aca2-3Galb1-4GlcNAcb1-2Mana1-6(Neu5Aca2-3Galb1-4GlcNAcb1-2Mana1-3)Manb1-4GlcNAcb1-4(Fuca1-6)GlcNAcb-Sp24	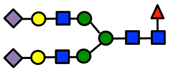	4566	74	2	20
25	Fuca1-2Galb1-4GlcNAcb1-2Mana1-6(Fuca1-2Galb1-4GlcNAcb1-2Mana1-3)Manb1/9-4GlcNAcb1-4(Fuca1-6)GlcNAcb-Sp22	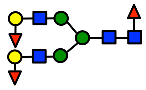	4339	533	12	570
26	GlcNAcb1-6(GlcNAcb1-2)Mana1-6(GlcNAcb1-4)(GlcNAcb1-4(GlcNAcb1-2)Mana1-3)Manb1-4GlcNAcb1-4(Fuca1-6)GlcNAc-Sp21	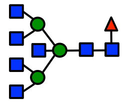	4091	236	6	14
27	Gala1-3(Fuca1-2)Galb1-4GlcNAcb1-2Mana1-6(Gala1-3(Fuca1-2)Galb1-4GlcNAcb1-2Mana1-3)Manb1-4GlcNAcb1-4(Fuca1-6)GlcNAcb-Sp22	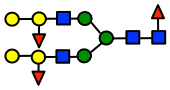	3649	529	15	564
28	Mana1-6(Mana1-3)Manb1-4GlcNAcb1-4(Fuca1-6)GlcNAcb-Sp19	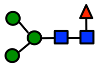	3630	244	7	25
29	Fuca1-2Galb1-3(Fuca1-4)GlcNAcb1-2Mana1-6(Fuca1-2Galb1-3(Fuca1-4)GlcNAcb1-2Mana1-3)Manb1-4GlcNAcb1-4(Fuca1-6)GlcNAcb1-4(Fuca1-6)GlcNAcb-Sp19	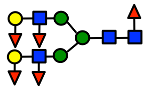	3222	144	4	120
30	Galb1-4GlcNAcb1-2Mana1-6(Galb1-4GlcNAcb1-2Mana1-3)Manb1-4GlcNAcb1-4(Fuca1-6)GlcNAcb-Sp22	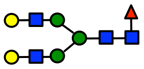	3222	524	16	19
31	Galb1-4GlcNAcb1-2 Mana1-6(GlcNAcb1-4)(Galb1-4GlcNAcb1-2Mana1-3)Manb1-4GlcNAcb1-4(Fuca1-6)GlcNAc-Sp21	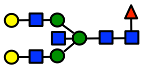	3216	143	4	29
32	GalNAca1-3(Fuca1-2)Galb1-3GlcNAcb1-2Mana1-6(GalNAca1-3(Fuca1-2)Galb1-3GlcNAcb1-2Mana1-3)Manb1-4GlcNAcb1-4(Fuca1-6)GlcNAcb-Sp22	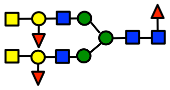	2820	454	16	418
33	Fuca1-2Galb1-3GlcNAcb1-2Mana1-6(Fuca1-2Galb1-3GlcNAcb1-2Mana1-3)Manb1-4GlcNAcb1-4(Fuca1-6)GlcNAcb-Sp22	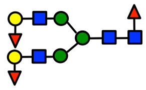	2614	126	5	421
34	Galb1-3GlcNAcb1-2Mana1-6(Galb1-3GlcNAcb1-2Mana1-3)Manb1-4GlcNAcb1-4(Fuca1-6)GlcNAcb-Sp22	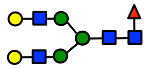	2390	426	18	130
35	Gala1-3(Fuca1-2)Galb1-3GlcNAcb1-2Mana1-6(Gala1-3(Fuca1-2)Galb1-3GlcNAcb1-2Mana1-3)Manb1-4GlcNAcb1-4(Fuca1-6)GlcNAcb-Sp22	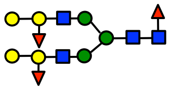	2381	293	12	n/a
36	GlcNAcb1-2Mana1-6(GlcNAcb1-2Mana1-3)Manb1-4GlcNAcb1-4(Fuca1-6)GlcNAcb-Sp22	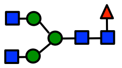	1708	194	11	26
37	GalNAca1-3(Fuca1-2)Galb1-4GlcNAcb1-2Mana1-6(GalNAca1-3(Fuca1-2)Galb1-4GlcNAcb1-2Mana1-3)Manb1-4GlcNAcb1-4(Fuca1-6)GlcNAcb-Sp22	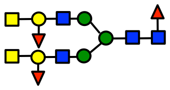	1558	87	6	511

41	Galb1-4(Fuca1-3)GlcNAcb1-2Mana1-6(Galb1-4(Fuca1-3)GlcNAcb1-2Mana1-3)Manb1-4GlcNAcb1-4(Fuca1-6)GlcNAcb-Sp22	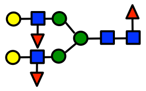	1069	24	2	287
46	Galb1-3GlcNAcb1-3Galb1-4GlcNAcb1-6(Galb1-3GlcNAcb1-3Galb1-4GlcNAb1-2)Mana1-6(Galb1-3GlcNAcb1-3Galb1-4GlcNAcb1-2Mana1-3)Manb1-4GlcNAcb1-4(Fuca1-6)GlcNAcb-Sp24	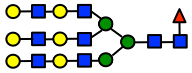	743	77	10	n/a
120	Fuca1-4(Galb1-3)GlcNAcb1-2Mana1-6(Fuca1-4(Galb1-3)GlcNAcb1-2Mana1-3)Manb1-4GlcNAcb1-4(Fuca1-6)GlcNAcb-Sp22	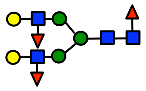	94	7	7	n/a

* GlcNAc, N-Acetyl-D-glucosamine; Man, D-Mannose; Neu5Ac, N-Acetylneuraminic acid; GalNAc, N-Acetyl-D-galactosamine; Gal, D-Galactose; Fuc, D-Fucose

** GlcNAc: blue square; Man, green circle; Neu5Ac: purple diamond; GalNAc: yellow square; Gal: yellow circle; Fuc: red triangle

Table S3. **rIgG core-fucosylated N-glycans: enrichment efficiency.** UPLC-HILIC-FLR profiles of unbound (FT) and lectin-bound (EL) fractions (including profiles of BKF-treated fractions) were integrated using Empower 3 software, and the enrichment efficiency was calculated using the obtained relative areas of each core-fucosylated glycan peak in FT (+ lectin), EL (+ lectin) and FT (no lectin) fractions.

Retention time (min)	GU	N-glycan composition	Capture efficiency (% of total)
4.64	5.82	HexNAc(4)Hex(3)dHex(1)	91
5.26	6.19	HexNAc(5)Hex(3)dHex(1)	93.1
6.03	6.6	HexNAc(4)Hex(4)dHex(1)	91.7
6.25	6.73	HexNAc(4)Hex(4)dHex(1)	92.5
6.51	6.87	HexNAc(5)Hex(4)dHex(1)	93.4
6.78	6.99	HexNAc(5)Hex(4)dHex(1)	94
7.76	7.51	HexNAc(4)Hex(5)dHex(1)	97.9
8.07	7.68	HexNAc(5)Hex(5)dHex(1)	96.6
9.42	8.38	HexNAc(4)Hex(4)Neu5Gc(1)dHex(1)<br>HexNAc(5)Hex(4)Neu5Gc(1)dHex(1)	91
10.9	9.17	HexNAc(4)Hex(5)Neu5Gc(1)dHex(1)	91.2
11.42	9.42	HexNAc(5)Hex(5)dHex(1)Neu5Gc(1)	94.6
14.04	10.8	HexNAc(4)Hex(5)dHex(1)Neu5Gc(2)	97.8
14.29	10.97	HexNAc(5)Hex(5)dHex(1)Neu5Gc(2)	91.5

Table S4. hIgG core-fucosylated N-glycans: enrichment efficiency. UPLC-HILIC-FLR profiles of unbound (FT) and lectin-bound (EL) fractions (including profiles of BKF-treated fractions) were integrated using Empower 3 software, and the enrichment efficiency was calculated using the obtained relative areas of each core-fucosylated glycan peak in FT (+ lectin), EL (+ lectin) and FT (no lectin) fractions.

Retention time (min)	GU	N-glycan composition	Capture efficiency (% of total)
4.64	5.85	HexNAc(4)Hex(3)dHex(1)	87
5.26	6.21	HexNAc(5)Hex(3)dHex(1)	96.8
6.03	6.64	HexNAc(4)Hex(4)dHex(1)	89.3
6.25	6.76	HexNAc(4)Hex(4)dHex(1)	88.5
6.51	6.89	HexNAc(5)Hex(4)dHex(1)	87.2
6.78	7.01	HexNAc(5)Hex(4)dHex(1)	87
7.76	7.54	HexNAc(4)Hex(5)dHex(1)<br>HexNAc(3)Hex(4)Neu5Ac(1)dHex(1)	91.1
8.07	7.71	HexNAc(5)Hex(5)dHex(1)	87
8.56	7.96	HexNAc(4)Hex(4)Neu5Ac(1)dHex(1)<br>HexNAc(5)Hex(4)Neu5Ac(1)dHex(1)	89.4
9.22	8.32	HexNAc(5)Hex(4)Neu5Ac(1)dHex(1)	96.7
10.07	8.75	HexNAc(4)Hex(5)dHex(1)Neu5Ac(1)	92.7
10.55	9.01	HexNAc(5)Hex(5)dHex(1)Neu5Ac(1)	93.8
12.37	9.93	HexNAc(4)Hex(5)dHex(1)Neu5Ac(2)	96.9
12.65	10.07	HexNAc(5)Hex(5)dHex(1)Neu5Ac(2)	94.5

```
AGP58216      1    ------MLTGTFLGLGALALAATAVAPAANA-----APVPVTSLNCDGDTYTCTAGLRFGDGRWVAQWNVNVFHQSPA--  67
WP_037947329  1    MS-aRRMLTGTFLGLGALALAATAVAPAANA-----APVPVTGLNCDGDTYKCTANLRFGDGRWVAQWNVNVFHQSAA--  72
WP_059148527  1    MS-aRRMLTGTFLGLGALALAATAVAPAANA-----APVPVTALNCDGDTYKCTANLAFGDGRWVAQWNVNVFHQSAA--  72
WP_030825832  1    MS-aRRMLTGTFLGLGALALAATAVAPAANA-----APVPVTALTCDGDTYKCTANLAFGDGRWVAQWNVNVFHQSAA--  72
WP_062012936  1    MS-aRRMLTGTFLGLGALALAATAVAPAANA-----APVPVTALTCDGDTYKCTANLAFGDGRWVAQWNANVFHQSAA--  72
WP_055550715  1    MS-aRRMLTGTFLGLGALLMVSTAVAPAATA-----APVPVTTLNCDGDTYKCTAGLRFGDGRWFADWNANVFHQSAArs  74
WP_018088730  1    M---RRTIVGTCAALGALAIGLAG---PATA-----APVPVTALNCDGDTYQCTANLRFGDNNWKASWGANIFHQTPR--  67
KJA18763      1    MQ-----LTNSFLSLLAAAQAIVAVPFGSSHvtntlAPVPVTKLVCDGNSFKCTASLDFGDGNWVAQWGTAVFHTGLFg-  74
WP_042158013  1    M---RRTIVGTCAALGALAIGLAG---PATA-----APVPVTALNCDGDTYQCTANLRFGDNNWKASWGANIFHQTPR--  67
WP_030083396  1    M---RRTIVGTCAAFAALGIGLAG---PAVA-----APVPVTALNCDGDTYKCTANLRFGDNNWKAGWGANIFHQAAR--  67
WP_030983681  1    M---RRTIVGTCAAFVAWGIGLAG---PAVA-----APVPVTALNCDGDTYKCTANLRFGDNNWRAGWGANIFHQVTR--  67
WP_033267611  1    M---RRTIAGICAALGALGIGGAA---PASA-----APVPVTALHCDGDTFTCTASLRFGDNNWKAGWSANVYHQAVR--  67
WP_051772026  1    MT-fKKALIALVVMASAPVFAGSAVAEPTPRd-----PVPVTGLWCDGDTYKCTAALDFGDGGWVADWSADVYH-EDRa-  72
BAL02930      1    MN-----FTASFLALLAAAQSIAAFPFGTTGaktalAPVPVTKLTCDGASFMCTANLDFADGNWVAQWTSPVFHRGNFg-  74
WP_006601771  1    M---RRTIVGTCAALAVLGIGSAA---PAVA-----APVPVTALNCDGDTYKCTANLRFGDNNWKARWDASIFHQANR--  67
WP_054290187  1    -----------------------------------------MSCDGDTYRCTAALDFGDGGWVADWSANVYH-A-Rv-  34
WP_015801300  1    MNitTKRIAAATSLAAASLLAAVV---PASAd-----PVDASGLTCDGQTYECVANLDFGDGNWTASWGAGVMHVDDLa-  71

AGP58216      68   sqqraarsadapRGAAAPVPVTTLTCDGDTYKCTAGLQFGDGRWVAQWNANVFHQAMANAVPTasaktvkaprEAAAPVP  147
WP_037947329  73   shqrvtrsadapRGAAAPVPVTTLTCDGDTYKCTAGLQFGDGRWVAQWNANVFHQAVASAVPAtsakavkaprEAAAPVP  152
WP_059148527  73   shqratrsadalRSAAAPVPVTTLTCDGDTYKCTAGLQFGDGRWVAQWNANVFHQAVTNAVPAasaktvkaprEAAAPVP  152
WP_030825832  73   shqratgsadalRTAAAPVPVTTLTCDGDTYKCTAGLRFGDGRWVAQWNANVFHQAMAHAESAasaktvkaprEAAAPVP  152
WP_062012936  73   srqratgsadalRTAAAPVPVTSLTCDGDTYKCTAGLRFGDGRWVAQWNANVFHQAMAHAVSAasaktvkaprEAAAPVP  152
WP_055550715  75   asaqsaesaqsaESVRAPVPVTTLNCDGDTYKCTAGLRFGDGRWVAQWDARVFHQA--------gaqamkaprGEAAPVP  146
WP_018088730  68   ------------SAYRAPVPVTALNCDGDTGKCIANLQFGDGYWRASWSANIFHQAFRSADGA----------RLAAPVP  125
KJA18763      75   ---apdhkefmhEMTLAPVPVTKLVCDGDTFQCTANLDFGDGRWVAQWSTSVFHNSLRSETTH-----------L-APVP  139
WP_042158013  68   ------------SASRAPVPVTALNCDGDTGKCIANLQFGDGYWRASWSATIFHQGLRSGDGT----------RLAAPVP  125
WP_030083396  68   ------------SAYRAPVPVTGLSCDGDTGKCVANLQFGDGYWRASWPATIFHQALRSADGA----------RLAAPVP  125
WP_030983681  68   ------------SAYRAPVPATSLSCDGDTGKCVANLQFGDGYWRASWPATIFHQALRSADGA----------RLAAPVP  125
WP_033267611  68   ------------SAYRAPVPATSLSCDGDTGQCVAGLQFGDGRWRASWPANVFHQALRSADGA----------RLAAPVP  125
WP_051772026  73   -------------QQRDMVPVTRLRCDGDTYTCTANLRFGDGRWVASWSVDVYRTG--RAVQL-------------DPVE  124
BAL02930      75   ---apdhkdiieQMAMAPVPVTELTCDGSSFLCTANLDFGDGNWVAQWPANVFHSGFHPGGRK-----------LFAPVP  140
WP_006601771  68   ------------SASRAPVPATSLNCDGDNGTCVADLMFGDGRWRASWPATVFHPALRSAEG-----------RLVAPVP  124
WP_054290187  35   -------------EPRAMVPVTRLRCDGDTYKCTANLKFGDGRWVADWSVRVHHTN--RAEPR-------------SPVR  86
WP_015801300  72   -------------QQRSPVGVTKVTCDGDTWTCTANLDFGDGGWTAEWGAWIAHEDKDEDARE-------------APVD  125

AGP58216      148  VTALNCDGDTYKCTAGLQFGDGRWVAQWDASVFHQS--------  183
WP_037947329  153  VTALNCDGDTYKCTANLQFGDGRWVAQWDASVFHQN--------  188
WP_059148527  153  VTALNCDGDTYKCTANLGFGDGRWVAQWDASVFHQN--------  188
WP_030825832  153  VTALNCDGDTYKCTAGLQFGDGRWVAQWDASVFHQN--------  188
WP_062012936  153  VTALNCDGDTYKCTANLQFGDGRWVAQWDASVFHQN--------  188
WP_055550715  147  VTALNCDGDTGLCTANLQFGDGRWAAQWGARIFHQ---------  181
WP_018088730  126  ANSLNCDGNIGRCSVNLQFGDGNWKANWPATIYHL---------  160
KJA18763      140  VTKLVCDGDSFKCTGSLDFGDGKWVAQWSTSVFHQSaffleqk  182
WP_042158013  126  ANSLNCDGNIGRCSVNLQFGDGNWKANWPATIYHL---------  160
WP_030083396  126  ANSLNCDGDTGRCSVNLQFGDGNWKADWPATIYHQ---------  160
WP_030983681  126  ANSLNCDGDTGRCSVNLQFGDGNWKADWPATIYHQ---------  160
WP_033267611  126  ANSLNCDGDTGRCSVNLQYGDGNWKAGWPVTVYHQG--------  161
WP_051772026  125  VTGLWCDGDTYKCKAALDFGDGDWVADWSADIFHT---------  159
BAL02930      141  VTDLNCDGETFMCTGNLDFADGNWKAQWNTNVFHNNnffkqn-  182
WP_006601771  125  ANSLNCDGDTGRCSVNLQFGDGGWKAAWAATIYHQP--------  160
WP_054290187  87   VTGLWCDGDTYKCKAALDFGDGRWVADWNVKVYHS---------  121
WP_015801300  126  VYELTCDGDEGKCVAKLDFGDGRWAAEWSARIKHS---------  160
```

Figure S1. Multiple sequence alignment of the hypothetical proteins identified through similarity search. Aligned protein sequences are from *Streptomyces rapamycinicus* NRRL 5491 (AGP58216); *Streptomyces sp.* PRh5 (WP_037947329); *Streptomyces violaceusniger* (WP_059148527); *Streptomyces hygroscopicus* (WP_030825832); *Streptomyces sporocinereus* (WP_062012936); *Streptomyces sp.* NBRC 110028 (WP_055550715); *Streptomyces sp.* FxanaC1 (WP_018088730); *Hypholoma sublateritium* FD-334 SS-4 (KJA18763); *Streptomyces sp.* NBRC 110027 (WP_042158013); *Streptomyces decoyicus* (WP_030083396); *Streptomyces sp.* NRRL S-1813 (WP_030983681); *Streptomyces lydicus* (WP_033267611); *Saccharothrix sp.* NRRL B-16314 (WP_051772026); *Pholiota nameko* (BAL02930); *Streptomyces auratus* (WP_006601771); *Kibdelosporangium phytohabitans* (WP_054290187) and *Actinosynnema mirum* (WP_015801300).

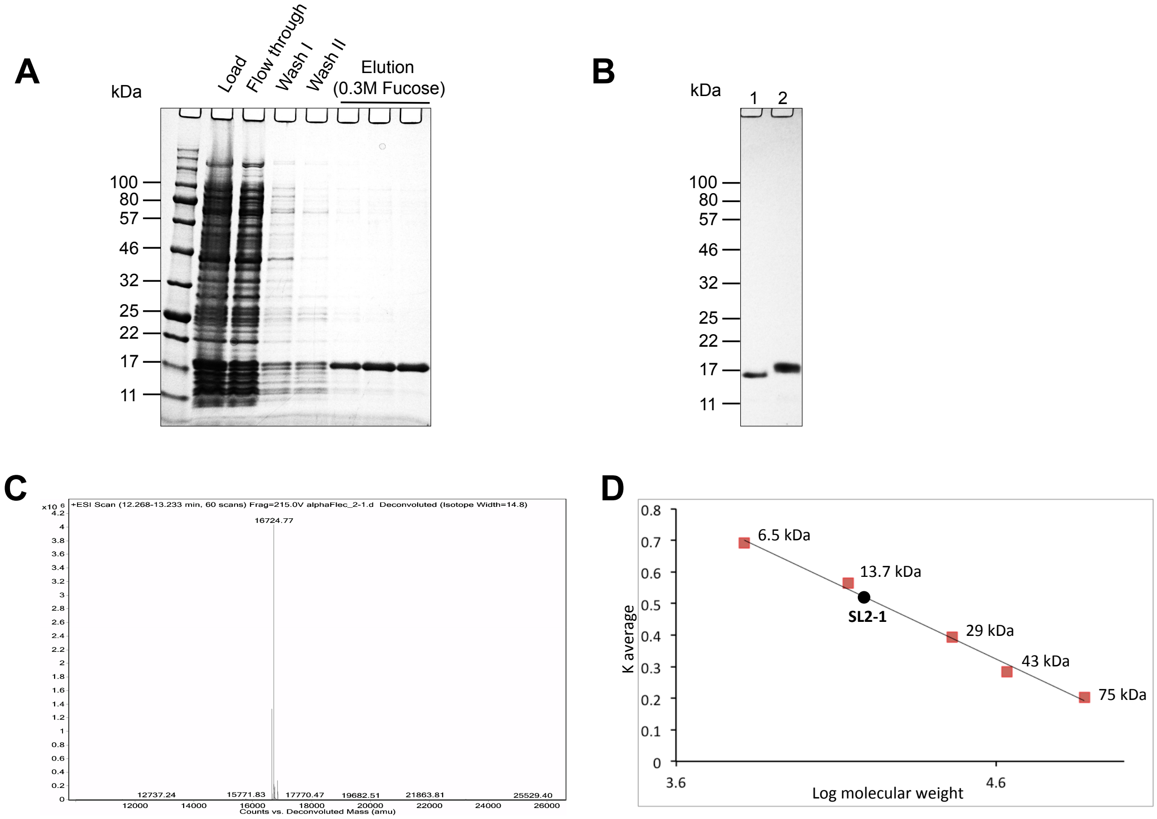

Figure S2. Expression, purification and characterization of recombinant SL2-1. (A) Recombinant protein was expressed in *E. coli* and purified by affinity chromatography using α-L-fucose agarose resin. (B) Non-reduced (lane 1) and reduced (lane 2) purified protein detected as single band of ~17 kDa on the denaturing polyacrylamide gel. (C) Electrospray ionization mass spectrometry of recombinant SL2-1. (D) The apparent molecular mass of the native SL2-1 was estimated by gel filtration on a HiPrep 16/60 Sephacryl S-200 HR column.

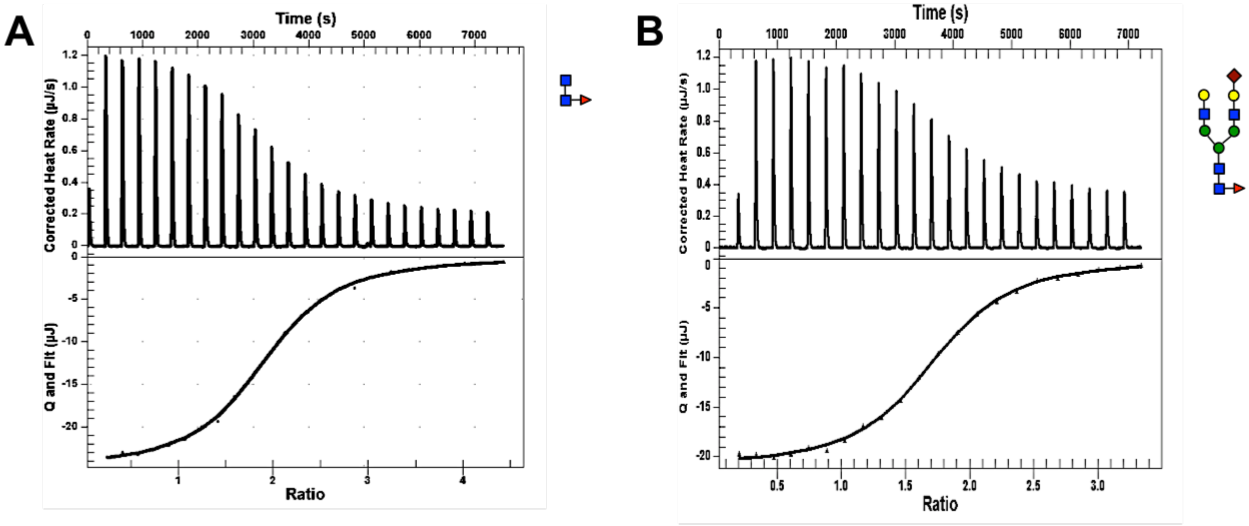

Figure S3. Typical ITC titration curves obtained for α1-6 fucosylated chitobiose (N2F) and a monosialo-fucosylated biantennary N-glycan (A1F).

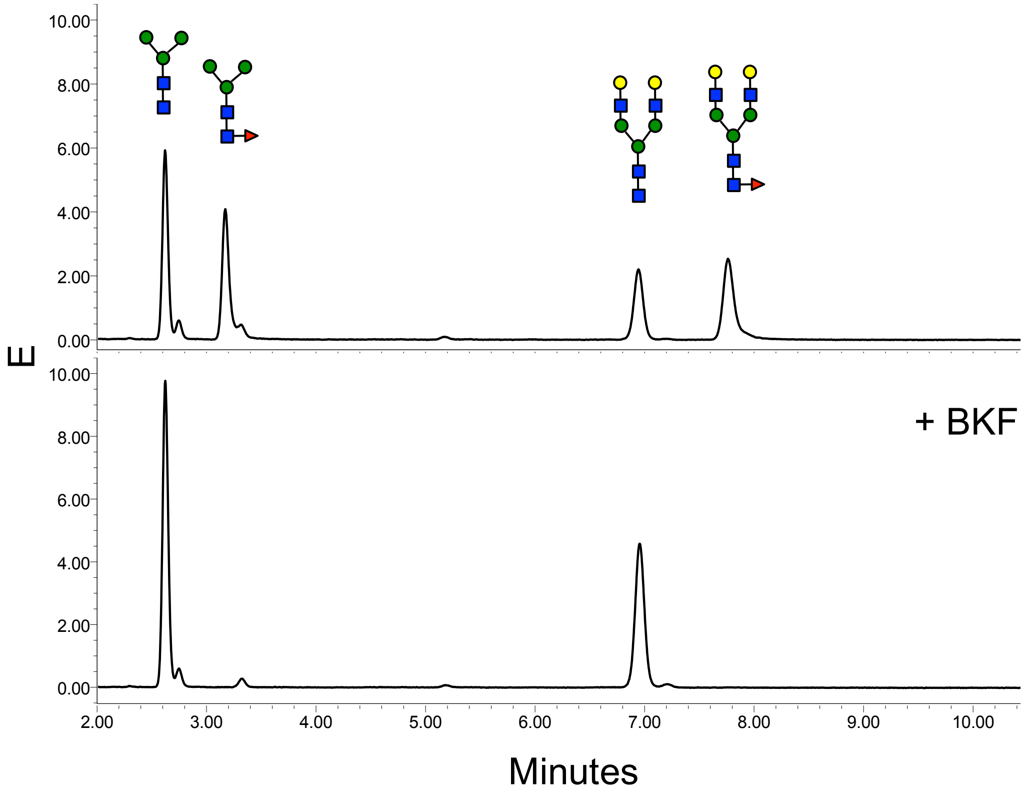

Figure S4. **UPLC-HILIC-FLR profiles of undigested vs BKF-digested M3N2, M3N2F, NA2 and NA2F N-glycans.**

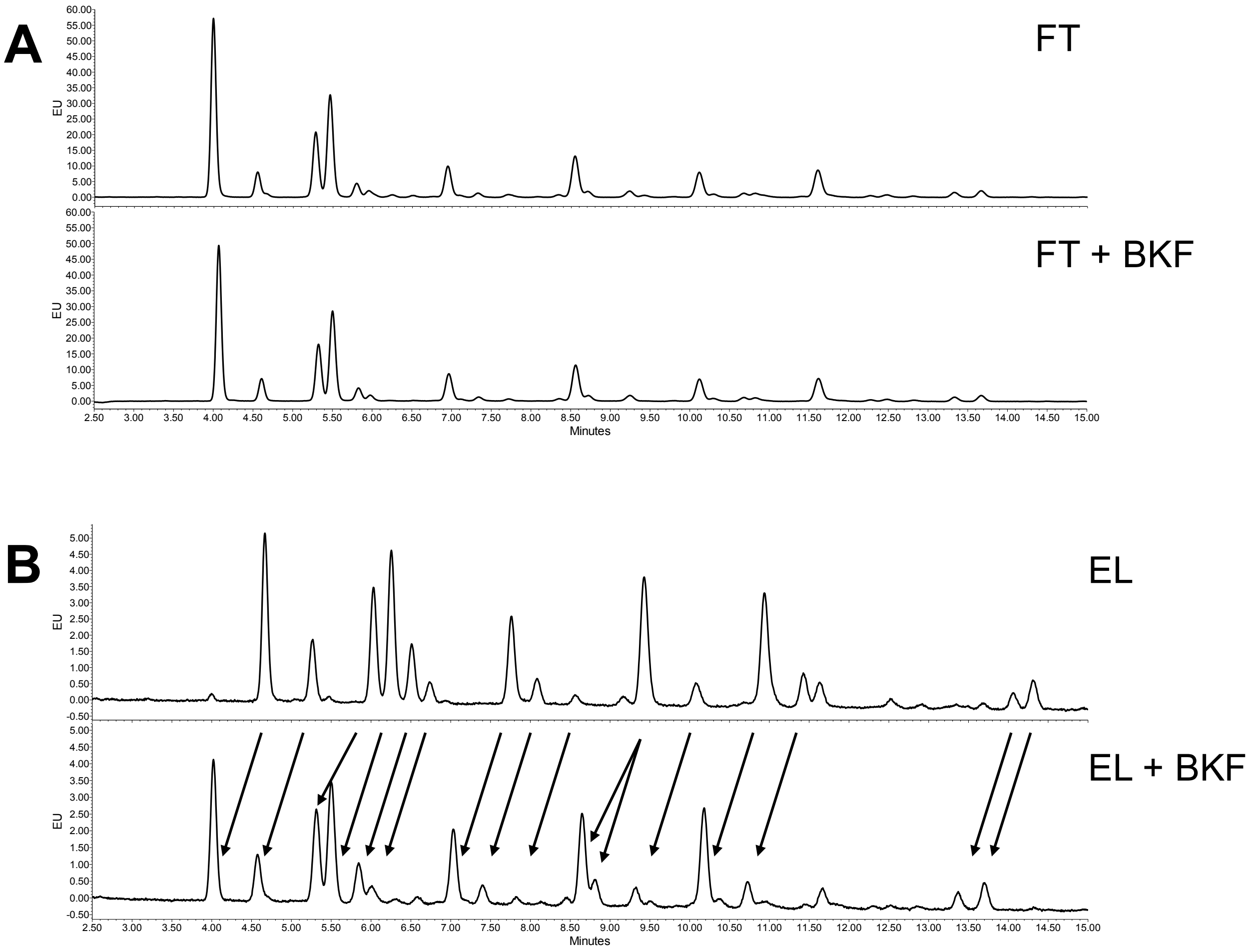

Figure S5. **UPLC-HILIC-FLR profiles of undigested vs BKF-digested N-glycans from rabbit serum IgG.** Unbound (FT) (A) and lectin-bound (EL) (B) fractions were treated with α1-2,4,6 fucosidase (BKF) and analyzed by Glycan BEH Amide HILIC chromatographic separation.

Figure S6. **Structural identification of N-glycans from rabbit serum IgG by mass spectrometry.** Identified carbohydrate compositions, observed masses and their sensitivity to BKF treatment provided in the table below (core-fucosylated structures labeled in red; non-fucosylated – in black).

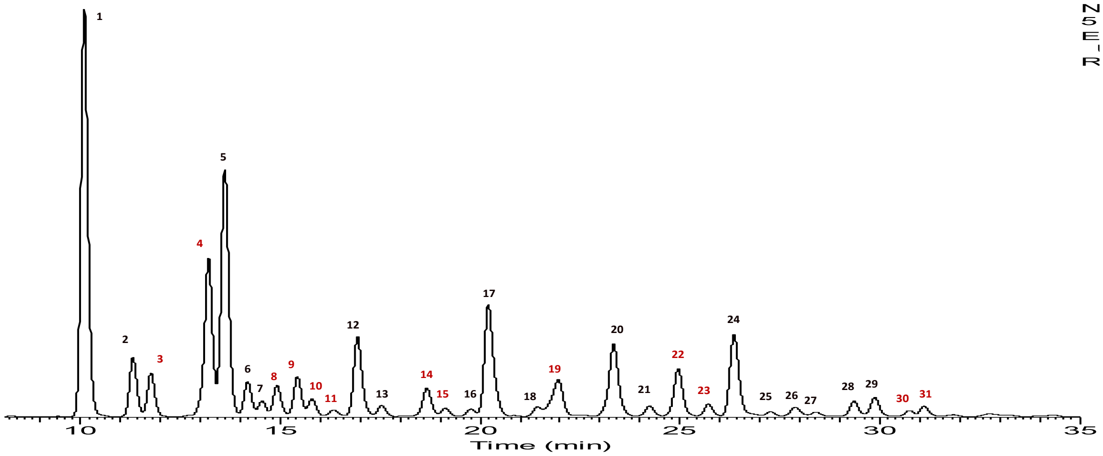

Peak #	Composition	m/z [+1]‡ theor.	m/z [+1]‡ observed	BKF-sensitive
1	HexNAc(4)Hex(3)	1437.5625	1437.65	-
2	HexNAc(5)Hex(3)	1640.6419	1640.66	-
3	HexNAc(4)Hex(3)dHex(1)	1583.6205	1583.65	+
4	HexNAc(4)Hex(4)<br>HexNAc(5)Hex(3)dHex(1)	1599.6154<br>1786.6998	1599.66<br>1786.75	-<br>+
5	HexNAc(4)Hex(4)	1599.6154	1599.66	-
6	HexNAc(5)Hex(4)	1802.6947	1802.73	-
7	HexNAc(5)Hex(4)	1802.6947	1802.73	-
8	HexNAc(4)Hex(4)dHex(1)	873.3403*	873.36*	+
9	HexNAc(4)Hex(4)dHex(1)	873.3403*	873.36*	+
10	HexNAc(5)Hex(4)dHex(1)	974.88*	974.95*	+
11	HexNAc(5)Hex(4)dHex(1)	974.88*	974.95*	+
12	HexNAc(4)Hex(5)	1761.6682	1761.75	-
13	HexNAc(5)Hex(5)	1964.7476	1964.84	-
14	HexNAc(4)Hex(5)dHex(1)	954.367*	954.37*	+
15	HexNAc(5)Hex(5)dHex(1)	1055.9064*	1055.96*	+
16	?	?	1055.46*	-
17	HexNAc(4)Hex(4)Neu5Gc(1)	953.8565*	953.87*	-
18	HexNAc(5)Hex(4)Neu5Gc(1)	1055.3962*	1055.46*	-
19	HexNAc(4)Hex(4)Neu5Gc(1)dHex(1)<br>HexNAc(5)Hex(4)Neu5Gc(1)dHex(1)	1026.8854*<br>1128.4251*	1026.95*<br>1128.46*	+<br>+
20	HexNAc(4)Hex(5)Neu5Gc(1)	1034.8829*	1034.87*	-
21	HexNAc(5)Hex(5)Neu5Gc(1)	1136.4226*	1136.42*	-
22	HexNAc(4)Hex(5)Neu5Gc(1)dHex(1)<br>HexNAc(4)Hex(5)Neu5Ac(2)	1107.9119*<br>1172.4332*	1107.96*<br>1172.46*	+<br>-
23	HexNAc(5)Hex(5)Neu5Gc(1)dHex(1)	1209.4515*	1209.47*	+
24	HexNAc(4)Hex(5)Neu5Ac(2)	1172.4332*	1172.46*	-
25	HexNAc(5)Hex(6)Neu5Gc(1)	1217.449*	1217.47*	-
26	HexNAc(4)Hex(5)Neu5Ac(1)Neu5Gc(1)	1180.4306*	1180.46*	-
27	HexNAc(5)Hex(5)Neu5Ac(1)Neu5Gc(1)	1281.9703*	1281.97*	-
28	HexNAc(4)Hex(5)Neu5Gc(2)	1188.4281*	1188.46*	-
29	HexNAc(5)Hex(5)Neu5Gc(2)	1289.9678*	1289.97*	-
30	HexNAc(4)Hex(5)Neu5Gc(2)dHex(1)	1261.457*	1261.47*	+
31	HexNAc(5)Hex(5)Neu5Gc(2)dHex(1)	1362.9967*	1362.97*	+

‡2AB-labeled glycans
*m/z [+2]

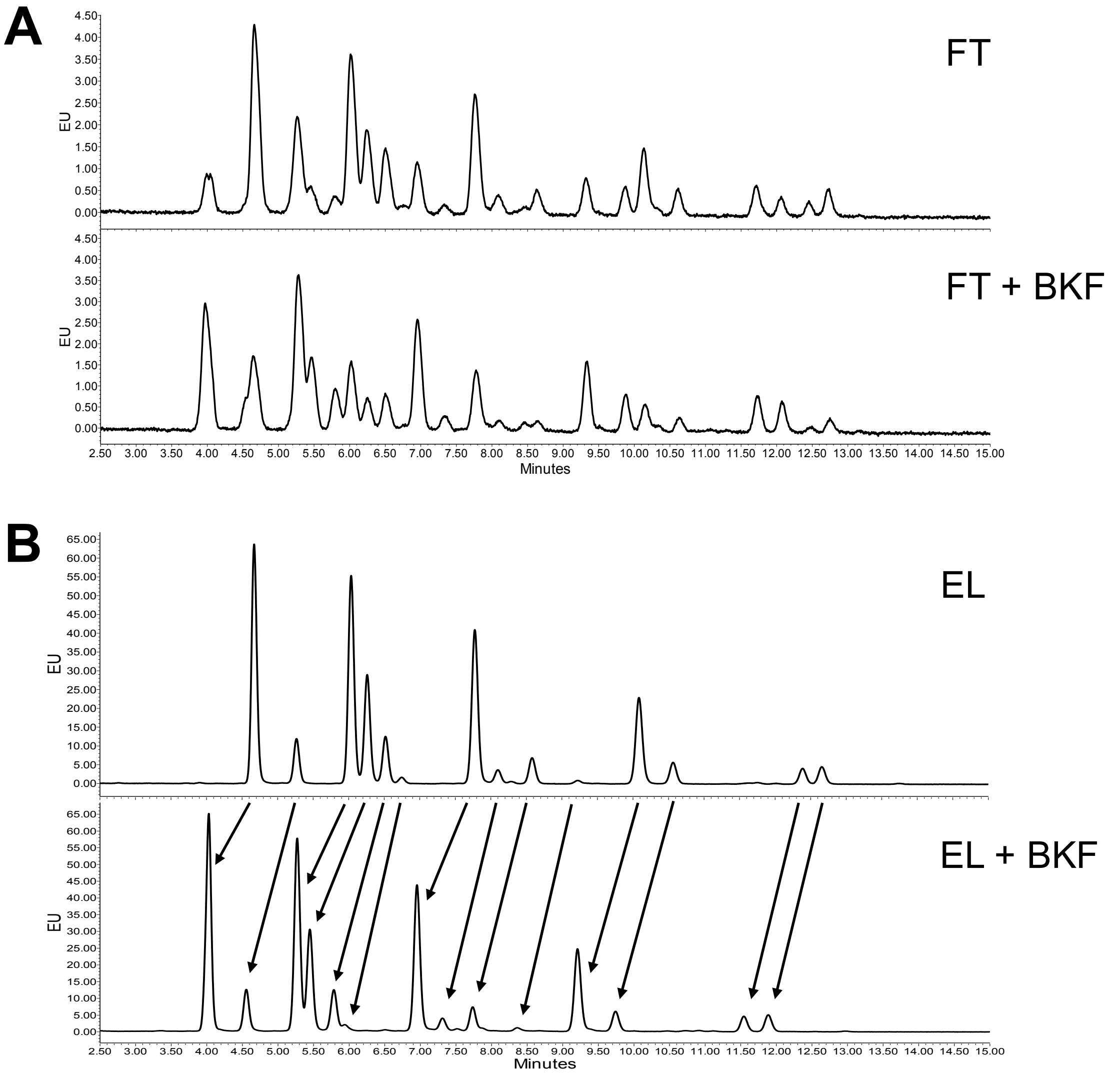

Figure S7. **UPLC-HILIC-FLR profiles of BKF-digested vs undigested N-glycans from human serum IgG.** (Unbound (FT) (A) and lectin-bound (EL) (B) fractions were treated with α1-2,4,6 fucosidase (BKF) and analyzed by Glycan BEH Amide HILIC chromatographic separation.

Figure S8. Structural identification of N-glycans from human serum IgG by mass spectrometry. Identified carbohydrate compositions, observed masses and their sensitivity to BKF treatment provided in the table below (core-fucosylated structures labeled in red; non-fucosylated – in black).

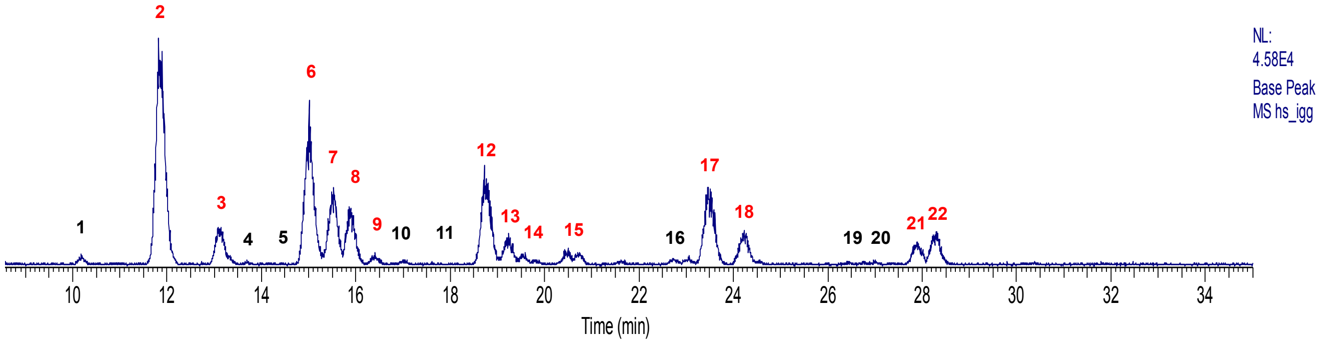

Peak #	Composition	m/z [+1]‡ theor.	m/z [+1]‡ observed	BKF-sensitive
1	HexNAc(4)Hex(3)	1437.5625	1437.65	-
2	HexNAc(4)Hex(3)dHex(1)<br>HexNAc(5)Hex(3)	1583.6205<br>1640.6419	1583.65<br>1640.66	+<br>-
3	HexNAc(5)Hex(3)dHex(1)	1786.6998	1786.75	+
	HexNAc(4)Hex(4)	1599.6154	1599.66	-
4	HexNAc(4)Hex(4)	1599.6154	1599.66	-
5	HexNAc(5)Hex(4)	1802.6947	1802.75	-
6	HexNAc(4)Hex(4)dHex(1)<br>HexNAc(5)Hex(4)	1745.6733<br>1802.6947	1745.75<br>1802.75	+<br>-
7	HexNAc(4)Hex(4)dHex(1)	1745.6733	1745.75	+
8	HexNAc(5)Hex(4)dHex(1)	1948.7527	1948.84	+
9	HexNAc(5)Hex(4)dHex(1)	1948.7527	1948.68	+
10	HexNAc(4)Hex(5)	1761.6682	1761.75	-
11	HexNAc(5)Hex(5)	982.8774*	982.87*	-
12	HexNAc(4)Hex(5)dHex(1)<br>HexNAc(3)Hex(4)Neu5Ac(1)dHex(1)<br>HexNAc(5)Hex(4)Neu5Ac(1)	1907.7261<br>917.3483*<br>1047.3987*	1907.76<br>917.36*<br>1047.37*	+<br>+<br>-
13	HexNAc(5)Hex(5)dHex(1)<br>HexNAc(5)Hex(4)Neu5Ac(1)	1055.9064*<br>1047.3987*	1055.96*<br>1047.46*	+<br>-
14	HexNAc(4)Hex(4)Neu5Ac(1)dHex(1)<br>HexNAc(5)Hex(4)Neu5Ac(1)dHex(1)	1018.8880*<br>1120.4277*	1018.87*<br>1120.46*	+<br>+
15	HexNAc(5)Hex(4)Neu5Ac(1)dHex(1)<br>HexNAc(4)Hex(5)Neu5Ac(1)	1120.4277*<br>1026.8854*	1120.46*<br>1026.87*	+<br>-
16	HexNAc(5)Hex(5)Neu5Ac(1)	1128.4251*	1128.46*	-
17	HexNAc(4)Hex(5)Neu5Ac(1)dHex(1)	1099.9144*	1099.96*	+
18	HexNAc(5)Hex(5)Neu5Ac(1)dHex(1)	1201.4541*	1201.46*	+
19	HexNAc(4)Hex(5)Neu5Ac(2)	1172.4332*	1172.38*	-
20	HexNAc(5)Hex(5)Neu5Ac(2)	1273.9728*	1273.97*	-
21	HexNAc(4)Hex(5)Neu5Ac(2)dHex(1)	1245.4621*	1245.47*	+
22	HexNAc(5)Hex(5)Neu5Ac(2)dHex(1)	1347.0018*	1347.06*	+

‡2AB-labeled glycans ,
*m/z [+2]
